# The gut microbiota profile of adults with kidney disease and kidney stones: a systematic review of the literature

**DOI:** 10.1186/s12882-020-01805-w

**Published:** 2020-06-05

**Authors:** Jordan Stanford, Karen Charlton, Anita Stefoska-Needham, Rukayat Ibrahim, Kelly Lambert

**Affiliations:** 1 University of Wollongong, School of Medicine, Faculty of Science, Medicine and Health, Wollongong, New South Wales 2522 Australia; 2 Illawarra Health and Medical Research Institute, Wollongong, New South Wales 2522 Australia; 3 University of Wollongong, Health Impacts Research Cluster, Wollongong, New South Wales 2522 Australia; 4 University of Surrey, School of Biosciences and Medicine, Faculty of Health and Medical Sciences, Guildford, GU2 7XH UK

**Keywords:** Gastrointestinal microbiome, Kidney diseases, Nephrolithiasis, Systematic review, Diet therapy

## Abstract

**Background:**

There is mounting evidence that individuals with kidney disease and kidney stones have an abnormal gut microbiota composition. No studies to date have summarised the evidence to categorise how the gut microbiota profile of these individuals may differ from controls. Synthesis of this evidence is essential to inform future clinical trials. This systematic review aims to characterise differences of the gut microbial community in adults with kidney disease and kidney stones, as well as to describe the functional capacity of the gut microbiota and reporting of diet as a confounder in these studies.

**Methods:**

Included studies were those that investigated the gut microbial community in adults with kidney disease or kidney stones and compared this to the profile of controls. Six scientific databases (CINHAL, Medline, PubMed, Scopus, Web of Science and Cochrane Library), as well as selected grey literature sources, were searched. Quality assessment was undertaken independently by three authors. The system of evidence level criteria was employed to quantitatively evaluate the alteration of microbiota by strictly considering the number, methodological quality and consistency of the findings. Additional findings relating to altered functions of the gut microbiota, dietary intakes and dietary methodologies used were qualitatively summarised.

**Results:**

Twenty-five articles met the eligibility criteria and included data from a total of 892 adults with kidney disease or kidney stones and 1400 controls. Compared to controls, adults with kidney disease had increased abundances of several microbes including *Enterobacteriaceae, Streptococcaceae, Streptococcus* and decreased abundances of *Prevotellaceae, Prevotella, Prevotella 9* and *Roseburia* among other taxa. Adults with kidney stones also had an altered microbial composition with variations to *Bacteroides, Lachnospiraceae NK4A136 group, Ruminiclostridium 5 group*, *Dorea, Enterobacter, Christensenellaceae* and its genus *Christensenellaceae R7 group*. Differences in the functional potential of the microbial community between controls and adults with kidney disease or kidney stones were also identified. Only three of the 25 articles presented dietary data, and of these studies, only two used a valid dietary assessment method.

**Conclusions:**

The gut microbiota profile of adults with kidney disease and kidney stones differs from controls. Future study designs should include adequate reporting of important confounders such as dietary intake to assist with interpretation of findings.

## Background

The link between the gut microbiome and human diseases continues to emerge in recent literature and has become a focus for global scientific endeavours to mitigate kidney disease development and progression [1]. The human microbiome is a complex ecosystem comprising of all the genetic material inside the trillions of microorganisms that live within and on us. Whereas, the microbiota is the community of micro-organisms found in a particular sample or location, which includes bacteria, bacteriophage, viruses, fungi, and protozoa [2, 3]. Advancements in microbial characterisation methods have facilitated our understanding of the complex mechanistic links between the microbiome and disease. There are numerous methods to categorise gut microbial composition and measure diversity. For instance, some methods are based on sequence divergences of the small subunit ribosomal RNA (16S rRNA) [4, 5], which provide helpful insights into the diversity of the gut microbiota, as well as qualitative and quantitative information based on what microbes are present. Examples of this technique include fluorescence in situ hybridization (FISH), DNA microarrays, and next-generation sequencing of the 16S rRNA gene or its amplicons [5]. Metagenomic or shotgun sequencing is another type of analysis which randomly sequences all extracted DNA in a given sample. This technique offers a higher taxonomic resolution by allowing the identification of microbial taxa present in a community at the species and strain level, in addition to providing information on the functional potential of the microbial community [4–6].

The microbiota encompasses far more metabolic genes than that of the human genome [2] and offers humans with additional unique functional capabilities via specific enzymes and metabolic pathways. Typically, these microbes have a harmonious relationship with their host [7] and contribute to several functional activities including micronutrient and immune homeostasis, energy metabolism, and host defences against pathogens [2, 8, 9]. However, in individuals with chronic kidney disease (CKD), evidence suggests that a microbial imbalance (dysbiosis) leads to an increase in harmful nephrovascular uraemic toxins [10–13]. The retention of these uraemic toxins, particularly indoxyl sulphate (IS), p-Cresyl sulphate (pCS), phenylacetylglutamine (PAG) and trimethylamine N-oxide (TMAO) have been associated with adverse complications, all of which negatively impact on the quality of life for individuals living with kidney disease. These complications include accelerated disease progression [14, 15], increased risk of cardiovascular-related mortality [15–17] and common symptoms such as constipation and cognitive decline [18]. Currently, the only known mechanism for reducing uraemic toxins in CKD is dialysis. Nevertheless, existing evidence indicates that only the free fraction of the protein-bound toxins pCS and IS can diffuse across the dialysis membrane, resulting in a limited capacity for removal [11]. Therefore, the development of novel strategies (other than dialysis) to reduce the production of major uraemic toxins, particularly in the earlier stages of CKD, is warranted.

The role of the gut microbiota in kidney stone disease historically focused on the presence of *Oxalobacter formigeme* [19], a gram-negative bacterium with the functional ability to degrade oxalate. Thus, a deficiency of *Oxalobacter formigene* present in stool samples was previously assumed to be a risk factor for kidney stone disease [20–22]. However, clinical studies provided questionable results, as *Oxalobacter formigene* has also been isolated from samples of recurrent stone formers [23, 24]. Since advancements in culture-independent methods for gut microbiota investigations that have become more available in the last decade [5], studies have made attempts to evaluate the relationship between gut microbiota and kidney stone formation in more comprehensive ways, shedding new light on the gut-kidney axis in nephrolithiasis.

Diet forms a critical component in the overall medical strategy for kidney disease and plays a fundamental role in determining the composition and functional activity of the human gut microbiota [25], with implications for uraemic toxin production [11, 13, 26–28]. Moreover, diet-based approaches such as ensuring adequate intake of fluid and dietary calcium, while avoiding high intakes of sodium and animal protein are crucial non-pharmacological prevention strategies for kidney stone disease. Therefore, dietary interventions and targeted nutritional therapies offer a potential approach to microbiota-associated diseases and mitigate uraemic toxin generation and kidney stone formation. Investigations into how the gut microbiota differs with disease and lifestyle factors such as diet will enhance not only our understandings of the contribution these microbes have in host biology but also understandings of the complicated exchange between diet and disease.

Differences in the composition of the gut microbiota in kidney disease and kidney stone populations compared to controls remains unclear due to the lack of a quantitative overview of existing evidence. The investigation into how the microbial signatures of adults with kidney disease or kidney stones may deviate from controls is essential to inform future trials. Hence, this review aims to systematically characterise the gut microbial composition in adults with kidney disease or kidney stones compared to controls and gain a better understanding of the functional capacity of the microbiota and reporting of diet as a confounder in these studies.

## Methods

### Protocol

The systematic literature review was reported following the Preferred Reporting Items for Systematic Reviews and Meta-Analyses (PRISMA) guidelines [29] and registered at PROSPERO (No. CRD42018109173, http://www.crd.york.ac.uk/).

### Article selection

The inclusion criteria were as follows:

1. Original research that examined the gut microbial community from adults with kidney disease or kidney stones and compared that to controls. This encompassed studies which reported to include adults with CKD; end-stage kidney disease (ESKD); glomerulonephritis; nephrotic syndrome; IgA nephropathy (IgAN); polycystic kidney disease; diabetic nephropathy (DN); Alport syndrome; Fabry disease; individuals who are receiving renal replacement therapies such as haemodialysis (HD), peritoneal dialysis (PD), kidney transplant (KT); or adults with kidney stones (any stone type).
2. Studies reporting on the microbial community from stool or intestinal biopsy samples;
3. Full-text articles available in English.

Studies for exclusion were:

1. Animal or in-vitro studies, case reports, abstracts, commentaries, review articles, editorials, expert opinion, letters, guidelines, protocols, seminars, reports, books or book chapters;
2. Study populations which included children;
3. Study populations which included adults with acute conditions (haemolytic uremic syndrome, acute kidney injury or urinary tract infections), renal cancer, kidney-yin deficiency syndrome or stones which were reported in other sites, such as the ureter or urethra.

### Search strategy and study selection

Six scientific databases including CINHAL, Medline, PubMed, Scopus, Web of Science and Cochrane Library as well as grey literature sources: Trove, the National Kidney Foundation Website, Google Scholar, Google.com.au, National Institute for Clinical Excellence (NICE) and TRIP Medical Database, were searched up until 7th August 2018. A research librarian was consulted to refine search terms and selection of databases. Reference lists of manuscripts were also scanned to discover additional relevant articles. A full-updated search using the original search strategy was undertaken on the 3rd October 2019 using the same six scientific databases as well as Google Scholar. The search strategy for all databases can be found in *Item 1* of this manuscript’s supplementary information (Additional file 1). All citations were imported by one member of the research team in Endnote (Endnote X8, Thomson Reuters, 2016) for review.

Two reviewers [JS, RI] independently screened the titles, abstracts and full texts for inclusion in this review. Discrepancies were resolved by consensus or adjudication by other members of the research team [KC, KL]. At the stage of full-text review, the decision to restrict articles to only those that included at least one of the following methods was made: Metagenomic sequencing (shotgun sequencing), amplicon-based sequencing methods including 16S rRNA sequencing, quantitative real-time polymerase chain reaction (qPCR) sequencing, DNA microarray or fluorescence in situ hybridization (FISH) techniques. These methods are culture-independent methods that enable phylogenetic identification and quantification, which will help to provide novel insights into the composition and/or diversity of the microbiota.

### Summary measures

The overall microbiota structure (α-diversity and β-diversity) and differences in the abundance of microbes at specific taxonomic levels (phylum, order, class, family, genus, species, OTU) were the primary outcomes of this paper. The secondary outcomes were descriptions of the real or predicted functional capacity of the microbiota, details on dietary intake and the dietary assessment methods used in each study.

### Data extraction and quality assessment

Relevant data in all eligible studies were extracted into an excel spreadsheet by one investigator, and another member of the research team cross-checked 25% of the input. Only statistically significant results (*p* < 0.05) that reported compositional differences of the gut microbiota between the groups of interest compared to controls, at each taxonomic level, were imported into excel. Additional information was also extracted: country of study, demographics (age, gender, and ethnicity), sample collection and storage methods, description of antibiotic and medication use, type of taxonomic database used, microbial characterisation method, microbial diversity metrics, and dietary methods.

To assess the quality of studies, three independent reviewers [JS, KL, ASN] used the Newcastle-Ottawa Scale (NOS) [30]. Appraisal items in the NOS tool were grouped into three categories: the selection of the study groups; the comparability of the groups; and the ascertainment of the exposure (the collection and assessment of samples for microbial communities) as the outcome of interest [30]. Overall, studies which scored seven or above using the NOS tool was considered to be of ‘high-quality’ [30–32].

### Data synthesis

Due to the heterogeneity of study methods and the absence of raw sequencing data, it was not possible to undertake a meta-analysis. Instead, a quantitative assessment of the microbial composition was performed. The quantitative approach applied the system of evidence level criteria (Table 1), according to a ranking-based system documented elsewhere [33–35]. Benefits of this approach are that it considers the number and methodological quality of included studies, as well as the consistency of reported findings. Study findings were deemed highly consistent and graded as ‘strong evidence’ when 75% or more of the studies that reported a particular bacteria were in agreement, whereby the same trend in microbial alteration (increased or decreased) relative to controls was found in at least two high-quality studies as determined by the NOS quality score. Finally, data on the functional potential of the microbial community, dietary intakes and dietary assessment methodologies used were qualitatively summarised.

**Table 1:** The system of evidence level criteria

Grading	Criteria
Strong	Consistent findings (≥75%) in at least 2 high-quality studies
Moderate	Consistent findings (≥75%) in one high-quality study and at least one low-quality study
Weak	Findings of one high-quality study or consistent findings (≥75%) in at least 3 or more low-quality studies
Inconclusive	Inconsistent findings, or consistent findings (≥75%) reported in less than 3 low-quality studies

## Results

### Summary of included studies

**Fig. 1:**
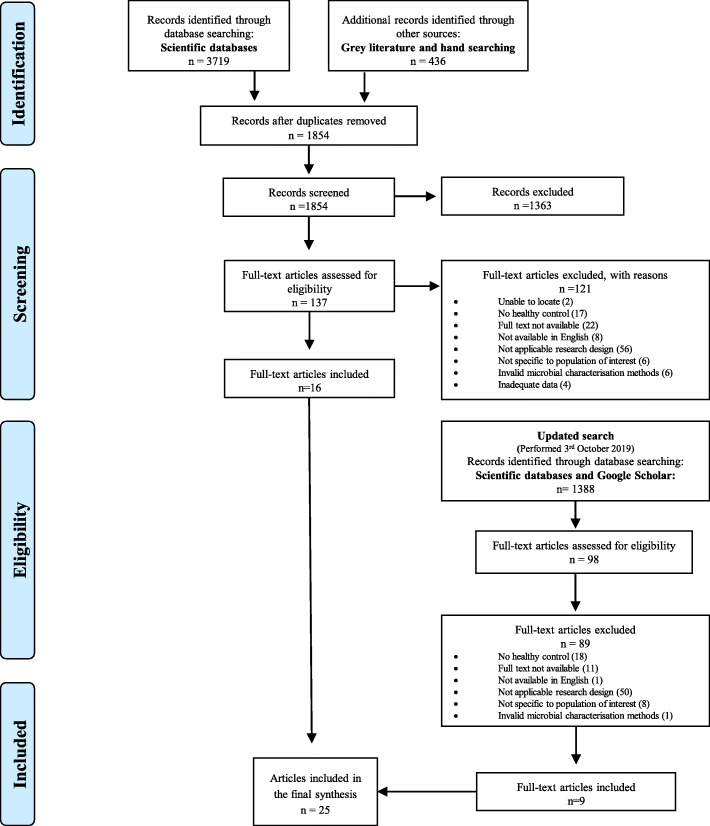
PRISMA flow diagram

The original literature search yielded 4155 articles, of which 110 full-text articles were evaluated. The updated search provided an additional 1388 papers, of which nine full-text articles were suitable for inclusion. A total of 25 articles met the eligibility criteria (Fig. 1). All 25 papers reported cross-sectional data on 722 adults with kidney disease, 170 adults with kidney stones and 1400 control participants (Table 2). Only different results obtained from the papers using the same study populations [36–38, 48] were extracted to avoid duplication.

**Table 2:** Characteristics of included studies

Reference	Country	Demographics of patients	Demographics of controls	Stool collection, temp. & storage	Antibiotic use	Medications	Taxonomy database	Microbiota analysis technique	Diversity metrics	Dietary assessment
**ADULTS WITH KIDNEY STONES (*n*= 6 articles)**										
Ticinesi 2018 [50]	Italy	*N* = 52 recurrent idiopathic calcium stones Age: 48 ± 11yrs, 60% male; ethnicity not reported	*N* = 48 Age: 47 ± 13yrs, 58% male; ethnicity not reported.	Self-sampled; Delivered to clinic within 2 hours from collection and stored at -22°C; Processed ≤ 2 weeks of receipt	Excluded if taken in prior 30 days.	Not reported	SILVA (16S rRNA), whole NCBI nr (shotgun)	16S ribosomal RNA gene sequencing (V3 region) Illumina MiSeq & shotgun metagenomics on sub-sample (*n*=10) Illumina NextSeq	↓ α-diversity in KS group vs. controls (Chao1 index; *p*<0.05); β-diversity significantly different between groups (*p*=0.002 with PERMANOVA and *p*=0.004 with Adonis test based on Bray-Curtis and weighted UniFrac metrics)	EPIC FFQ delivered by a nutritionist; Calcium intake lower in adults with KS (*p*<0.04). Calcium intake considered as a covariate in the analysis of gut microbiota data.
Stern 2016 [58]	USA	*N* = 23; all stone types included Age: 53.7 ± 15.4yrs, 22% male; 13% African American, 35% Hispanic, 52% Caucasian.	*N* = 6 Age: 53.5 ± 16.0yrs, 67% male; 50% African American and 50% Caucasian.	Self-sampled; Flash-frozen with dry ice & stored -80°C	Excluded if taken in prior 2 weeks.	Not reported	Greengenes	16S ribosomal RNA gene sequencing (V4 region) Illumina MiSeq	Not reported	Not reported
Tang 2018 [53]	China	*N* = 13; stone types not reported. Age: 52.25 ± 7.25yrs, 38% male; 100% Chinese.	*N* = 13 Age: 55.81 ±5.79yrs, 38% male; 100% Chinese.	Self-sampled; Immediately frozen & stored -80°C. (DNA samples stored at -20°C)	Control group did not have antibiotics in prior 3 months; unclear if this was the same for kidney stone group.	None of the adults with kidney stones were reported to be on any medications before sample collection.	RDP	16S ribosomal RNA amplicon sequencing (V4 region) Illumina Hiseq 2500 platform	α-diversity reported not significantly different (Chao1, Shannon, Simpson’s and Good’s coverage indices, *p*>0.05) Overall, β-diversity was distinct but not significantly different (*p*=0.096 with ANOSIM and *p*=0.058 with MPRR based on Bray-Curtis distance metric)	Not reported
Suryavanshi 2016 [38]	India	*N* = 24 recurrent calcium oxalate stones. Age: 22-50yrs, 100% male; ethnicity not reported.	*N* = 15 Age: 22-52yrs, 100% male; ethnicity not reported.	Self-sampled; Stored -80°C	Excluded if taken in prior 3 months.	Not reported	Greengenes (16S rRNA) & M5NR database (*frc*-gene)	16S ribosomal RNA gene and *frc-*gene amplicon sequencing; quantitative polymerase chain reaction & PCR-DGGE (V3 region of 16S rRNA gene) Iron Torrent PGM system	α-diversity reported not significantly different (Chao1, observed species/OTUs, phylogenetic diversity, Shannon and Simpson’s indices) β-diversity metrics reported to reveal compositional differences between groups (PCoA plots of weighted and unweighted UniFrac results; statistical test and *p*-value not reported).	Reported number of participants who follow a vegetarian diet; dietary intake results and assessment methods not reported.
Suryavanshi 2018 [37]	India	*Same as the population above* *N* = 24 recurrent calcium oxalate stones. Age: 22-50yrs, 100% male; ethnicity not reported.	*N* = 48 (Additional *n* = 33 controls recruited). Age and gender only reported for a subset of *n* =15 (as above); ethnicity not reported.	Not reported	Not reported	Not reported	Greengenes	Targeted sequencing (*frc-, but- and buk-*, 16S and 18S ribosomal RNA genes as well as fungal ITS1 region) & quantitative polymerase chain reaction Iron Torrent PGM system	α-diversity investigated in selected sub-sample (*n* = 7 KSD, *n* = 7 controls); authors reported significant decrease observed in KSD group (Chao1, Shannon and Simpson’s indices; *p*-value not reported). β-diversity not reported.	Not reported
Tavasoli 2019 [49]	Iran	*N* = 58 (majority confirmed to have calcium stones) *n* = 29 recurrent stones with hyperoxaluria Age: 44.21 ± 9.58yrs, 62.1% male. *n* = 29 recurrent stones without hyperoxaluria Age: 48.07 ± 9.20yrs, 72.4% male. Ethnicity not reported	*N* = 29 Age: 41.42 ± 11.48yrs, 89.7 % male; ethnicity not reported.	Self-sampled; stored -20°C	Excluded if taken in prior 2 months.	Medications not reported; individuals excluded from the study if taking calcium, magnesium, potassium or pyridoxine supplementation.	N/A	Real-time polymerase chain reaction	N/A	Not reported
**ADULTS WITH KIDNEY DISEASE (*n*=19 articles)**										
***Individuals not reported to be undertaking a renal replacement therapy (n=10)***										
Al-Obaide 2017 [42]	USA	*N* = 20 CKD* (CKD stage not reported, all were reported to have T2DM) eGFR: 16.54 ± 3.01; Age: 64.4 ± 2.3yrs. Gender and ethnicity not reported. *Stool samples of n=18/20 were analysed to identify the gut microbial profile	*N* = 20 Age:54.3±3.2yrs; eGFR, gender and ethnicity not reported.	Self-sampled; Processed ≤24hrs of receipt	Excluded if taken for atleast 3 consecutive days in the prior month	Participants of the T2DM-CKD group treated with insulin, OHAs, ranitidine, PPI, ACEi, ARBs, statin therapy	Curated database from Greengenes, RDPII & NCBI	16S ribosomal RNA gene sequencing (V3-V4 region) Illumina MiSeq	Not reported.	Dietary assessment methods not stated. Macronutrient data presented as a percentage of total energy intake; protein intake higher in controls (*p*<0.05) and fat intake higher in CKD group (*p*<0.01)
Barrios 2015 [61]	UK	*N* = 62 (CKD stage not reported). eGFR: reported ≤60; Age, gender and ethnicity not reported for sub-sample.	*N* = 793 eGFR >60; Age, gender and ethnicity not reported for sub-sample.	Self-sampled; Refrigerated up to 2 days prior to clinic visit; Stored at -80°C	Data on antibiotics usage was collected for the prior month; data available for only 11% of study participants.	Not reported	Greengenes	16S ribosomal RNA gene sequencing (V4 region) Illumina MiSeq	Not reported	Dietary scores were obtained from EPIC FFQ generated through PCA. Diet scores were considered as covariates in the linear mixed effects regression models; no significant results reported.
De Angelis 2014 [39]	Italy	*N* = 32 IgAN (CKD stage not reported) *n* = 16 non-progressors, eGFR: 76 ± 15; Age: 41 ± 10yrs, 69% males. *n*=16 progressors, eGFR: 30±18; Age:45 ± 6 yrs, 63% male. 100% Caucasian.	*N* = 16 eGFR: 96 ± 7; Age: 43 ± 8yrs, 60% male. 100% Caucasian	Self-sampled; Stored -80°C	Excluded if taken in prior 3 months.	All IgAN participants treated with ACEi. Other medications undefined; no remarkable changes reported to medications in prior month.	RDP	Bacterial tag-encoded FLX-titanium amplicon pyrosequencing bTEFTAP (V1-V3 region) 454 FLX Sequencer	↓ α-diversity in IgAN group vs. controls (Chao1, observed species/OTUs and Shannon indices; *p*<0.05) β-diversity not reported.	Reported no remarkable changes to participant’s diet in prior month. Dietary assessment method or results not reported.
Jiang 2016 [47]	China	*N* = 65 (CKD stage 1-5 ) eGFR: 55.61 ± 52.55; Age: 43.45 ± 16.90 yrs, 46% male. 100% Chinese	*N* = 20 eGFR: 104.99 ± 19.82; Age: 43.05 ± 9.88yrs, 30% male. 100% Chinese	Self-sampled; Stored at -80°C	Excluded if taken in prior 4 weeks.	Not reported	N/A	Quantitative polymerase chain reaction	N/A	Not reported
Xu 2017 [56]	China	*N* = 32 (CKD stages 4-5) eGFR: reported <30; Age: 53.34 ± 14.47yrs, 50% male; 100% Chinese	*N* = 32 eGFR: reported ≥90; Age: 55.03 ± 10.38yrs, 50% male; 100% Chinese	Sample collection method unclear; Stored at -40°C	Excluded if taken in the prior month.	Not reported	Not reported	16S rDNA and rRNA sequencing (V4 region) Illumina MiSeq	↓ α-diversity in CKD group vs. controls (phylogenetic diversity and Shannon indices; *p*<0.001) β-diversity significantly different between groups (ADONIS analysis based on unweighted UniFrac metric, *p*<0.001)	Not reported
Wang 2012 [44]	China	*N* = 30 (reported ESKD) Age: 54 (37-71) yrs, 53% male. eGFR and ethnicity not reported.	*N* = 10 Age: 55 (41-67yrs) yrs, 50% male. eGFR and ethnicity not reported.	Not reported	Excluded if taken in prior 3 weeks.	Medications not reported; Corticosteroids, statins, or cytotoxic drugs were not taken in the prior 3 weeks.	SILVA	16S ribosomal RNA gene pyrosequencing (V1-V3 region) Sequencing platform not reported	↑ α-diversity ESKD vs. controls (observed species/OTUs index; *p*=0.017). β-diversity not reported.	Not reported
Jiang 2017 [51]	China	*N* = 52 (CKD stage 5) eGFR: 6.86 ± 2.87; Age: 51.58 ± 18.33yrs, 56% male. 100% Han Chinese nationality.	*N* = 60 eGFR: 98.03 ± 27.32; Age:52.53 ± 13.98yrs, 42 % male. 100% Han nationally Chinese	Self-sampled; Stored at -80°C	Excluded if taken in prior 4 weeks.	ESKD participants treated with phosphate binders, anti-hypertensives and various vitamin and/or mineral supplementation (iron, calcium and vitamin D)	Not reported	Quantitative polymerase chain reaction & 16S ribosomal RNA gene pyrosequencing in sub-sample (*n* = 27 ESKD and *n* = 26 controls) (V4-V6 region) Illumina GAII	α-diversity reported not significantly different (Chao1, observed species/OTUs, Shannon and Simpson’s indices). No difference in β-diversity reported between groups (PCoA plot based on UniFrac metric, statistical test or *p*-value not reported).	Not reported
Gradisteanu 2019 [41]	Romania	*N* = 9 DN (CKD stage not reported) eGFR, age, gender and ethnicity not reported .	*N* = 5 eGFR, age, gender and ethnicity not reported.	Self-sampled; Stored at -20°C	Not reported	Not reported	N/A	Real-time polymerase chain reaction (Bacterial and fungal group-specific primers were also used)	N/A	Not reported
Tao 2019 [40]	China	*N* = 14 DN (CKD stage not reported) eGFR: 93.26 ± 17.0; Age:52.93 ± 9.98 yrs, 64% male. 100% Chinese.	*N* = 28 *n* = 14 healthy controls eGFR: 96.01 ± 9.29; Age: 52.86 ± 9.91yrs, 64% male. *n* = 14 T2DM controls without CKD eGFR: 93.48± 13.02; Age: 53.29yrs ± 9yrs, 64% male. 100% Chinese.	Self-sampled; stored at -80°C	Excluded if taken in the prior 30 days.	Not reported	SILVA	16S ribosomal RNA gene pyrosequencing (V3-V4 region) Illumina MiSeq	↑ α-diversity richness DN vs. T2DM controls (observed species/OTUs index, *p*= 0.023; but no difference found for other α-diversity indices) β-diversity significantly different between groups (PERMANOVA based on Bray-Curtis metric, *p* = 0.02).	Excluded if following restrictive diets. Reported similar/same eating habits across some of their groups; however, no formal dietary assessment was undertaken.
Li 2019 [52]	China	*N* = 50 (CKD stage not reported) eGFR: 22.39 ± 15.56; Age:52.4 ± 13.49yrs, 54% male. 100% Chinese.	*N* = 22 eGFR: 97.06 ± 12.98; Age:50.27 ± 7.77yrs, 55% male. 100% Chinese	Self-sampled; transported on ice and stored at -80°C	Excluded taken in prior 3 months.	Medications not reported; Immunosuppressive drugs not to be taken in prior 3 months.	RDP	16S ribosomal RNA gene pyrosequencing (V3-V4 region) Illumina HiSeq	↓ α-diversity in CKD group vs. controls (Phylogenic diversity whole-tree index), *p*<0.05, and reduced but not significant for Shannon or Simpson’s indices *p*> 0.05). β-diversity significantly different between groups (ANOSIM test based on unweighted UniFrac, *p*= 0.001)	Not reported
***Individuals undertaking a renal replacement therapy: Haemodialysis (n=3 articles)***										
Vaziri 2013 [48] & Wong 2014 [36]	USA	*N* = 24 eGFR not reported. Dialysis vintage: ≥3 months, Kt/V =1.5 ± 0.3. Age: 57 ± 14 yrs, 25% male. 38% Caucasian, 54% Hispanic, and 8% Asian.	*N* = 12 eGFR not reported. Age: 51 ± 12 yrs, 33% male. 33% Caucasian, 58% Hispanic, and 8% Asian.	Not reported	Excluded if taken in prior 3 months.	HD patients were treated with phosphate binders, ESAs (Darbopoetin), vitamin & mineral supplementation. Immunosuppressive drugs not to be taken in prior 3 months.	Greengenes (Wong)	Microarray sequencing; 16S ribosomal RNA gene PhyloChip analysis	Relative richness (assessed for subfamilies at subphylum level) was reported to be similar between groups (*p*-value not reported). β-diversity revealed tighter clustering in the control group than HD group (NMDS figure based on Bray-Curtis distance metric; statistical test or *p*-value not reported)	Strict fluid and dietary sodium, phosphorus, and potassium restrictions; Nutrition prescription, education or counselling methods and results not reported.
Miao 2018 [45]	China	*N* = 21 Dialysis vintage: ≥3 months, spKt/V reported >1.2 and regularly monitored. Age: 53.0 ± 9.0yrs, 57% male. eGFR and ethnicity not reported.	*N* = 20 (Unit staff) Age:31 ± 9.1yrs, 50% males. eGFR and ethnicity not reported.	Self-sampled; storage not reported.	Not reported	Medications at baseline not reported; HD patients had not taken lanthanum carbonate in prior 3 months.	RDP, BLAST	16S ribosomal RNA gene pyrosequencing (V1-V3 region) Sequencing platform not reported	α-diversity not significantly different (Shannon index; *p=*0.429). β-diversity not reported.	Diet reported being ‘controlled for’, although methods and results were not reported.
***Individuals undertaking a renal replacement therapy: Peritoneal dialysis (n=1 article)***										
Wang 2012 [43]	Taiwan	*N* = 29 eGFR: reported <15; Dialysis vintage: 49.7 ± 35.4 months, Kt/V not reported; Age: 53.7 ± 11.7yrs, 34% male. Ethnicity not reported.	*N* = 41 Age: 58.2 ± 12.8yrs, 37% male. eGFR and ethnicity not reported.	Self-sampled; Immediately put on ice; Processed ≤1hr of defecation	Excluded if taken in prior 30 days.	Not reported	N/A	Real-time polymerase chain reaction	N/A	Not reported
***Mixed cohorts of individuals with chronic and end-stage kidney disease including those undertaking different renal replacement therapies (n= 5 articles)***										
Shi 2014 [55]	China	*N* = 52 *n* = 22 HD group Dialysis vintage: 6-40 months, Kt/V not reported. *n* = 30 reported ESKD not undertaking dialysis. eGFR, age, gender and ethnicity not reported.	*N* = 10 eGFR, age, gender and ethnicity not reported.	Details of sample collection not reported; Samples immersed in 90% alcohol and stored at -20°C	Excluded if taken in prior 3 weeks.	Medications not reported; Corticosteroids, statins, cytotoxic drugs not taken in prior 3 weeks.	SILVA	16S ribosomal RNA gene Pyrosequencing (V1-V3 region) Sequencing platform not reported	↑ α-diversity in HD group vs. controls (average OTUs/ species, *p*=0.044) and Chao1 index higher in three HD samples (*p*-value not reported). β-diversity not reported between study groups. However, PCA plot of Unifrac data from selected samples at the genus level exhibited a large separation in the same three HD samples vs. the other selected samples (*p*-value or statistical test not report).	Not reported
Stadlbauer 2017 [57]	Austria	*N* = 30 *n* = 15 PD group GFR (ml/min): 7.9 (7.3; 14.0); Dialysis vintage: 25 (15-74) months. Age: 62yrs (54-69), 80% male. *n* = 15 HD group GFR (ml/min): 6.0 (5.9; 9.3); Dialysis vintage: 70 (40-197) months, Kt/V not reported. Age: 61yrs (54-71), 67% male; Ethnicity not reported.	*N* = 21 GFR (ml/min): 77.6 (73.4; 86.6). Age: 58 yrs (53-62), 43% male. Ethnicity not reported.	Self-sampled; Stored at -80°C	Not reported	Participants from HD and PD groups treated with phosphate binders, PPI, immunosuppressive drugs. Small number of controls (*n*=2) also treated with PPI.	SILVA	16S ribosomal RNA gene sequencing (V1-V2 region) Illumina MiSeq	↓ α-diversity in PD and HD group compared to controls (Chao1 index, *p*<0.05, similar results reported for observed species/OTUs and phylogenic diversity) β-diversity significantly different between HD vs. controls *p*=0.012 and PD vs. controls *p*=0.003 (ANOSIM test based on Bray Curtis, weighted and unweighted UniFrac metrics)	Not reported
Lun 2019 [60]	China	*N* = 49 (*n* =13 treated with HD) Age: 54 ±14 yrs, 76% male; eGFRand ethnicity not reported.	*N* = 24 Age: 56 ± 9 yrs, 67% males; eGFR and ethnicity not reported.	Self-sampled; Stored at -80°C	Excluded taken in prior 3 months.	Not reported	Not reported	16S ribosomal RNA gene sequencing (V3-V4 region) Illumina HiSeq (PE250)	α-diversity not reported. β-diversity reported being distinct between CKD vs. controls; unclear if statistically significant (PCA plot based on the Euclidean distance & NMDS based on the UniFrac distance metric; statistical test or *p*-value not reported)	Not reported
Li 2019 [62]	China	*N* = 53 *n* = 29 HD group eGFR: 5.75 (4.35–8.26); Dialysis vintage and Kt/V not reported. Age: 54yrs (41.5-69), 59% male. *n* = 24 ESKD group (stage 5) not undertaking dialysis. eGFR: 5.33 (4.31–7.72); Age: 55.5 yrs (48.25-64.5), 50% male. Ethnicity not explicitly reported.	*N* = 69 eGFR: 126.07 (102.80–148.65); Age: 51yrs (39.5-64), 39 % male. Ethnicity not explicitly reported.	Collected in sterile 2mL tube on ice, containing pure ethanol and frozen within 30 minutes; Stored at -80°C	Excluded if taken in prior 4 weeks.	HD and CKD participants were treated with phosphate binders. Medication history of the last month was collected, but results were not reported.	Greengenes	16S ribosomal RNA gene sequencing (V1-V2 region) Illumina HiSeq 2500 system	↓ α-diversity in CKD and HD group vs. controls (Chao1, ACE and Shannon indices *p*<0.001). β-diversity reported being different between groups (PCoA plot presented data based on weighted and unweighted UniFrac metrics, ANOSIM test, *p*-value not reported)	Reported similar eating habits across the cohort, although methods and results were not reported.
Guirong 2018 [46]	China	*N* = 100 *n* = 16 KT recipients (sampling reported within the first month after transplantation) Age: 42.8±11.5yrs. *n* = 84 CKD group (stage 3-4) Age: 55.9±18.2 yrs. eGFR, gender and ethnicity not reported.	*N* = 53 Age: 54.7 ± 12.8yrs; eGFR, gender and ethnicity not reported.	Collection and storage not reported.	None taken in prior 3 months.	Not reported	Not reported	16S ribosomal RNA gene sequencing (V3 region) Ion Personal Genome Machine system	↓ α-diversity in RT and HD vs. controls (Chao1 index *p*<0.001). β-diversity reported being different between groups (PERMANOVA based on Bray-Curtis metric, *p*<0.01)	Not reported

Legend: *ESKD* End-stage kidney disease, *HD* Haemodialysis, *HC* Healthy controls, *PD* Peritoneal dialysis, *KS* Kidney stones, *KT* Kidney transplant, *DN* Diabetic nephropathy, *OTU* Operational Taxonomic Units, *Wt* Weight, *Ht* Height, *WC* Waist circumference, *KSD* Kidney stone disease, *T2DM* Type 2 diabetes mellitus, *PPI* Protein pump inhibitors, *ESA* Erythropoiesis-stimulating agents, *ACEi* Angiotensin-converting-enzyme inhibitors, *ARBs* Angiotensin II receptor blockers, *OHA* Oral hypoglycaemic agents, *eGFR* Estimated glomerular filtration rate (ml/min/1.73m^2^), *GFR* Glomerular filtration rate (ml/min), *vs*. versus; ↓= decreased, ↑= increased

Majority of articles reported on adults with CKD who were not receiving a renal replacement therapy, including studies that exclusively investigated IgAN [39] and DN cohorts [40, 41] (Table 2, *n* = 10). Three papers investigated adults undertaking HD, and another study included individuals receiving PD therapy. Five additional articles included mixed kidney disease cohorts within their sample. No studies reported to exclusively investigate the gut microbiota profile in individuals with glomerulonephritis, nephrotic syndrome, polycystic kidney disease, Alport syndrome, Fabry disease, and compared that to a control group. The remaining six articles investigated adults with kidney stones. The age structure of cohorts did vary; although there was some overlap across studies, including a mean sample population age between 41 and 64.5 years. The portion of males ranged from 22 to 100%. Overall, studies were predominately from China (*n* = 12/25), followed by the United States of America (*n* = 3/25) and Italy (*n* = 2/25).

All eligible studies used stools samples for their gut microbiota analysis. Eighteen articles reported using frozen stool samples for their analysis, where storage temperatures ranged from − 20 °C to − 80 °C. Others reported that samples were processed within 24 h of receipt [42] or kept on ice and processed within 1 h of defecation [43]. The remaining articles failed to provide sufficient details regarding the storage and processing methods utilised [36, 44–46, 48]. Four articles assessed the gut microbiota using only qPCR [41, 43, 47, 49]. The remaining studies assessed gut microbiota via high-throughput molecular approaches: Illumina platforms (MiSeq, NextSeq, HiSeq), Iron Torrent PGM system or bTEFAP using 454 FLX sequencer, DNA microarray analysis performed using PhyloChip assay. However, three papers did not report the sequencing platform used [44, 45, 55]. Among these studies, four employed a combination of microbial characterisation techniques in addition to 16S rRNA sequencing, including: shotgun sequencing [50]; qPCR [38, 37, 51]; denaturing gradient gel electrophoresis (DGGE) fingerprinting [38] as well as gene-targeted amplicon sequencing of functional genes involved in oxalate and butyrate metabolism (*frc-* [37, 38], *but*- and *buk- gene* [37]). Two research groups also utilised fungal specific primers for their qPCR analysis [41], as well as 18S rRNA and fungal ITS1 region primers for amplicon sequencing [37] to explore members of the microbial communities other than just bacteria.

### Risk of bias and heterogeneity

Using the Newcastle-Ottawa Scale, of the 25 final articles, 11 were considered high-quality with a score of seven or above (Table 3). There were multiple sources of heterogeneity among studies, including: the collection, handling and storage temperature of samples, DNA extraction method, the primer used for PCR, the variable region of the conserved genes (for instance the 16S rRNA gene) and the sequencing platform used. Also, some methods employed cannot evaluate the whole microbial community, such as qPCR, which can only detect selected microbes. Moreover, although there were some consistencies, many papers differed with their statistically analysis and presentation of the gut microbial data.

**Table 3:** Quality assessment of included articles (*n* = 25)

Reference	Sampling (4 points)	Confounders controlled (2 points)	Exposure (3 points)	Total rating (9 points)
[37]	– − − −	– −	+ + −	2
[60]	– − − −	+ −	+ + −	3
[44, 46]	+ − − −	– −	+ + +	4
[41]	– − − −	+ −	+ + +	4
[36, 48]	+ − − −	+ −	+ + +	5
[55]	+ + − −	– −	+ + +	5
[45]	+ − − +	– −	+ + +	5
[38, 39, 47, 51]	+ − − −	+ +	+ + +	6
[42]	+ + − −	+ −	+ + +	6
[43, 53, 56, 57, 58, 61]	+ + − −	+ +	+ + +	7
[40, 49, 52, 62]	+ − − +	+ +	+ + +	7
[50]	+ + + −	+ +	+ + +	8

Legend: ‘+’ Quality criterion satisfied; ‘-’ Quality criterion not satisfied or insufficient information to adjudicate as satisfied. Studies with a quality score of seven or above were considered high-quality

A few articles were also not consistent in reporting the CKD stage of their participants or details of the diagnostic criteria used for the classification of CKD. Other sources of heterogeneity included the age, gender, ethnicity of participants and the recruitment of controls. For instance, one study only recruited males [38], while seven of the eight papers that reported ethnicity recruited one ethnic group: Chinese [40, 47, 51–53, 56] or Caucasian [39]. Details relating to the recruitment of control groups were often missing or differed across studies, with some articles reporting that their controls were hospital or clinic staff. Furthermore, the classification of ‘controls’, the timing of antibiotic usage before study participation, reporting of medications and other co-existing conditions was not consistent across studies and more often poorly defined.

### Microbial diversity and richness

Alpha (α) diversity is the measure of the number (richness) and distribution (evenness) of taxa within a sample [54]. Sixteen articles reported results for α-diversity, of which five observed no significant difference between groups [38, 45, 48, 51, 53]. Eight articles reported that α-diversity was significantly reduced in adults with kidney disease or kidney stones relative to controls, among which seven papers reported a statistically signficant *p*-value [39, 46, 50, 52, 56, 57, 62]. Interestingly, the remaining three studies observed a significant increase in α-diversity (observed species/OTUs) in individuals with ESKD, DN and those undergoing HD therapy compared to controls [40, 44, 55].

Beta (β) diversity, a measure of the diversity that represents the similarity or difference in microbial composition between sites or different samples [54], was evaluated in 12 studies (using Bray-Curtis, weighted and unweighted UniFrac distance metrics). Ten studies reported compositional differences in the overall microbial communities between controls and kidney disease or kidney stone cohorts, of which six studies [40, 46, 50, 52, 56, 57] reported a statistically significant *p*-value (Table 2).

### Altered microbial composition

Alterations of the microbial composition were presented into two larger groups: adults with kidney disease and adults with kidney stones. To highlight alterations of the microbial composition specific to individuals receiving dialysis treatment, data from relevant articles that included adults undertaking HD or PD therapy are presented as a sub-analysis.

#### Microbiota profile of adults with kidney disease compared to controls

**Fig. 2:**
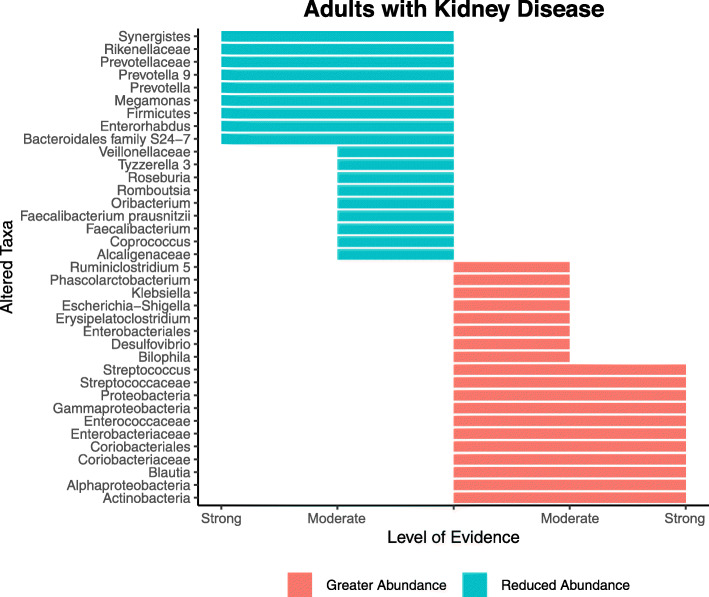
Altered taxa based on strong and moderate level of evidence for adults with kidney disease compared to controls. Figure includes data from studies that investigated adults with *CKD*, *IgAN*, *DN*, *ESKD*, *KT* recipients and individuals receiving dialysis therapy (*HD* and *PD*)

Figure 2 characterises the differences in the gut microbiota profile of individuals with kidney disease compared to controls. Based on strong level of evidence criteria, 20 microbial taxa were reported as being differentially abundant. For instance, adults with kidney disease had relatively increased abundances of Proteobacteria, *Enterobacteriaceae, Streptococcaceae, Streptococcus* and decreased abundances of Firmicutes, *Prevotellaceae, Prevotella,* and *Prevotella* 9. Based on the moderate evidence criteria, nine other taxa were less abundant in comparison to controls including *Alcaligenaceae, Roseburia, Faecalibacterium* and *Faecalibacterium prausnitzii*. In contrast, *Bilophila, Desulfovibrio, Klebsiella*, *Escherichia-Shigella,* along with four other taxa were more highly abundant (Fig. 2).

The population of an additional 112 microbial taxa were altered, but this was based on weak evidence (data not shown; Table S1, Additional file 1). Of these, 53 taxa were more abundant in individuals with kidney disease, while the remaining 59 were less abundant when compared to controls.

##### Microbiota profile of adults receiving dialysis therapy

**Fig. 3:**
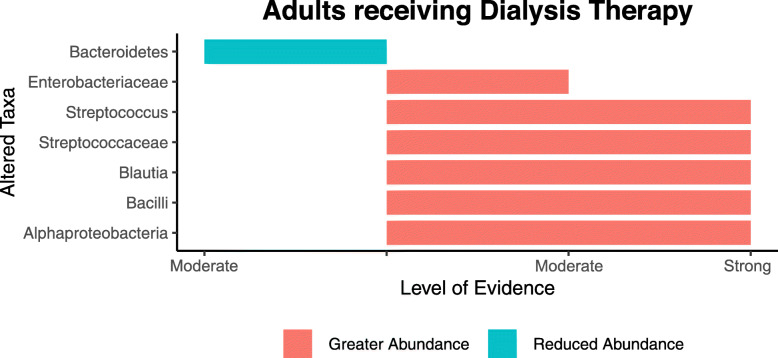
Sub-sample analysis of altered taxa based on strong and moderate level of evidence for adults with kidney disease receiving dialysis therapy compared to controls. Figure includes data only from the studies that investigated adults receiving *HD* or *PD* therapy

Data from the six articles included in this sub-analysis identified that five microbial taxa including Alphaproteobacteria, *Streptococcaceae,* and *Streptococcus* were more abundant in adults receiving dialysis than controls based on strong evidence criteria (Fig. 3). Based on moderate evidence criteria, the abundance of Bacteroidetes was reduced, while *Enterobacteriaceae* was higher in adults undergoing dialysis therapy. Results from two other studies [46, 60] were not included in this sub-analysis because it was unclear if the CKD cohort were receiving haemodialysis at the time of sample collection [46], and whether the results of individuals undertaking dialysis therapy were statistically significant compared to controls [60].

Stadlbauer et al. [57] investigated differences in the microbial profile according to dialysis type. Compared to controls, adults receiving PD had reduced abundances of *Comamonadaceae* and *Campylobacteraceae* and increased abundances of *Ruminococcaceae* and *Corpococcus.* In HD participants, microbial families *Comamonadaceae* and *Campylobacteraceae* were reportedly enriched, while *Faecalibacterium prausnitzii*, *Roseburia intestinalis* and *Clostridium nexile* were depleted compared to controls.

##### Microbiota profile of kidney transplant recipients

One study in this review [46] included a sub-set of adults who received a KT (*n* = 16) within the prior month of study commencement. Their findings revealed that 54 taxa were altered compared to controls: Firmicutes, *Faecalibacterium, Prevotella* and three other bacterial members were less abundance, while Bacteroidetes, Proteobacteria, Alphaproteobacteria, *Streptococcaceae, Streptococcus* along with 43 other taxa were enriched in individuals who received a KT (data not shown; Table S1, Additional file 1). Authors reported that based on the abundance of major phyla, the overall gut microbial structure of KT recipients was more similar to the participants with CKD (stages 3–4) rather than the controls [46]. However, several microbial taxa were unique to KT recipients. For example, Proteobacteria and Enterobacteriaceae were more highly abundant in KT recipients in comparison to the CKD cohort.

#### Microbiota profile of adults with kidney stones compared to controls

**Fig. 4:**
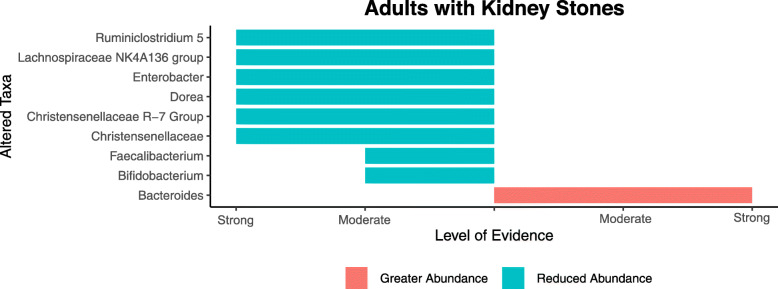
Altered taxa based on strong and moderate level of evidence for adults with kidney stones compared to controls

Based on strong evidence criteria, seven taxa were observed to be altered in adults with kidney stones. Within the phylum Firmicutes, *Lachnospiraceae NK4A136 group*, *Ruminiclostridium 5 group*, *Dorea*, *Christensenellaceae*, its genus *Christensenellaceae R7 group,* as well as *Enterobacter* (from the Proteobacteria phylum) were all significantly reduced compared to controls (Fig. 4). In contrast, *Bacteroides* was more highly abundant in adults with kidney stones. Finally, the abundance of two other taxa, *Bifidobacterium and Faecalibacterium* were found to be significantly reduced based on moderate evidence criteria in adults with kidney stones (Fig. 4).

The population of an additional 85 microbial taxa were altered in kidney stone populations compared to controls, but this was based on weak evidence (data not shown; Table S2,  Additional file 1), of which 32 microbes increased and 53 taxa decreased. Presence and absence data of archaea, microeukaryotes and fungi from Survyanshi et al. [37] were not included as it was unclear if results were statistically significant. Given this was the only paper that employed a high-throughput approach to examine different microbial community members (other than bacteria), it seemed relevant to summarise their findings in the manuscript's supplementary information (Table S3, Additional file 1).

#### Microbial markers for the potential detection of kidney disease and kidney stones

Six studies reported values of the area under the receiver operating characteristic (ROC) curve (AUC), ranging between 0 and 1 [59]. The higher AUC value, the better the test or model is at distinguishing between participants with disease and no disease [59]. Findings amongst these studies were inconclusive. 

Li et al. [52] identified *Akkermansia* (AUC = 0.753) and *Lactobacillus* (AUC = 0.792) was able to differentiate between adults with CKD and controls, while the combination of both genera achieved the best result (AUC = 0.830). Lun et al. [60] reported that *Lachnospira* (AUC = 0.813) performed best for controls, while *Ruminococcus gnavus* (AUC = 0.764) was best to detect adults with CKD. In another study [46], the presence of 4 genera from the bacterial family *Lachnospiraceae* (*Shuttleworthia, Pseudobutyrivibrio, Roseburia and Lachnospira*) were able to identify adults with CKD from controls with high accuracy (AUC = 0.92). The model by Tao et al. [40], which included *Escherichia Shigella* and *Prevotella 9,* had an AUC = 0.86 for predicting DN in their study population. In adults with kidney stones, Tavasoli et al. [49] reported that none of the bacteria examined in their study was able to generate an acceptable AUC to differentiate from controls, while Tang et al. [53] reported that *Pseudomonas aeruginosa* and *Escherichia coli* could be used to classify their nephrolithiasis patients accurately (AUC = 0.947 and AUC = 0.840, respectively).

### Altered genetic functions of the microbiota

Among nine studies, eight [37, 38, 40, 46, 50, 52, 56, 57] reported that the real or predicted functional capacity of the gut microbiota in adults with kidney disease and kidney stones were substantially different from controls. Only one of these study [50] undertook shotgun sequencing in a subset of their study population (*n* = 10 participants) to investigate the real functional potential of the microbial communities for known metabolic pathways. Functional gene-targeted amplicon sequencing was also employed by another research group [37, 38]. However, the majority of studies predicted the functional capacity of the gut microbiota using the bioinformatic software platform PICRUSt (Phylogenetic Investigation of Communities by Reconstruction of Unobserved States) [38, 40, 46, 52, 53, 56, 57].

In adults with stage 4–5 CKD, using PICRUSt, functional genes relating to trimethylamine (TMA) metabolism were increased (K07811, K07821, and K03532), while functional genes relevant to choline, betaine and L-carnitine metabolism (K07271, K01004, K00499, K00130 and K00540) were found to be significantly reduced compared to controls [56]. In another study investigating adults with CKD, microbial genes associated with the circulatory system function were predicted to be significantly enriched, while polyketide metabolism, as well as cell motility and secretion, was reduced relative to controls [52]. Similarly, Guirong et al. [46] also found that predicted microbial genes relevant to polyketides metabolism were also significantly reduced in adults with CKD and KT recipients along with genetic information processing (involving the ribosome, homologous recombination, and aminoacyl-tRNA), metabolism of co-factors, vitamins, nucleotides, terpenoids and cellular processes including pyrimidine metabolism. Compared to controls, KT recipients and adults with CKD had significantly greater predicted microbial genes associated with the metabolism of carbohydrates, other amino acids, and xenobiotics [46]. Differentially abundant bacterial functions related to lipid metabolism were reported between DN, T2DM controls and controls [40]. In adults undergoing dialysis (HD or PD), four functional pathways relevant to the renin-angiotensin system, glycosphingolipid biosynthesis, isoflavonoid biosynthesis and vasopressin regulated water reabsorption were different compared to controls [57]. However, the authors [57] did not report if these functional pathways were upregulated or downregulated.

In adults with kidney stones, shotgun sequencing analysis found that functional genes involved in oxalate degradation, such as formyl-CoA transferase and oxalyl-CoA decarboxylase, were significantly reduced compared to controls [50]. The highest representation of these genes was detected in some Archaea and Bacteria, either with known (*Oxalobacter formigenes*) or previously unknown oxalate-degrading properties (*Escherichia coli, Eggerthella spp., Roseburia hominis, Bacteroides massiliensis, Clostridium citroniae*) [50]. Through the application of DGGE fingerprinting and targeted-gene sequencing of the *frc-gene,* Suryavanshi et al. [38] also confirmed that in addition to *Oxalobacter formigenes,* several gut inhabitants possessed the ability to metabolise oxalate. However, Suryavanshi et al. [38] findings using PICRUSt completely contrasted the shotgun sequencing results [50], as several genes involved in oxalate degradation were reported to be enriched rather than depleted in their kidney stone cohort [38]: formate dehydrogenase (K08349), oxalate/formate antiporter (K08177), formyl-CoA transferase (K07749), oxalyl-CoA decarboxylase (K01577) and oxalate decarboxylase (K01569). Tang et al. [53], who also investigated these same metabolic pathways relevant to oxalate degradation, reported observing no significant difference between groups. Other predicted functional activities of the gut microbiota relating to energy metabolism, glycan synthesis, metabolism of co-factors and vitamins were down-regulated, while lipid metabolism, carbohydrate metabolism and xenobiotic degradation metabolism were upregulated in adults with kidney stones [38]. In more recent work [37], through targeted-gene sequencing of the *buk-gene,* numerous butyrate-producing bacterial species that were present in controls, were not found in samples from participants with kidney stones.

Wong et al. [36] employed a different approach to the above methods. This research group adopted a targeted approach using the Kyoto Encyclopedia of Genes and Genomes (KEGG) database as well as a literature review to search for corresponding functional genes of interest alongside the list of bacterial families that differed in relative abundance between adults receiving HD and controls [36]. In individuals undertaking HD therapy, 12 of the 19 microbial families that were of highest abundance were urease-possessing families, while an additional five families reportedly possessed the uricase gene (*Cellulomonadaceae, Dermabacteraceaea, Micrococcaceae, Polyangiaceae, Xanthomonadaceae*). Three bacterial families were believed to contain the tryptophanase gene *(Clostridiaceae, Enterobacteriaceae, and Verrucomicrobiaceae*) and four were suspected to be capable of deaminating tyrosine into p-Cresol, a precursor of pCS. Furthermore, two of three bacterial families (*Lactobacillaceae and Prevotellaceae*) that were less abundant in adults receiving HD have reported butyrate-producing functions, including members that possess phosphotransbutyrylase and butyrate kinase genes.

### Dietary intake assessment and methodologies

Three of the 25 articles (12%) presented dietary intake results, of which two considered diet in their analysis [50, 61]. The authors concluded that dietary factors did not seem to be involved in kidney disease− or nephrolithiasis−associated abnormalities of the gut microbial composition.

Of the three papers, two used the 131-item European Prospective Investigation of Cancer and Nutrition (EPIC) Food Frequency Questionnaire (FFQ) [50, 61], one of which employed a trained research nutritionist to administer the FFQ [50]. Barrios et al. [61] generated dietary scores from the FFQ results through principal component analysis [61]. These dietary scores were then considered as covariates in their analysis of the gut microbiota. In the second study, Ticinesi et al. [50] assessed total energy (kcal), macronutrient (protein, carbohydrates, fats in grams), dietary fibre (grams), alcohol (grams) and micronutrient consumption (grams) between groups (adults with kidney stones and controls). Only dietary calcium intake, along with other outcomes such as BMI, age, the sex were adjusted as covariates in microbiota analyses [50]. The third study presented macronutrient percentage consumption of total energy intakes for carbohydrates, proteins and fats between groups, but details relaying the methods used for data collection, or if the dietary data were considered in the analysis, were missing [42]. Other papers included in this review did not present results or assess actual dietary intakes. For instance, authors simply reported that their cohort groups had similar eating habits [40, 62], or noted which participants were self-reported vegetarians [38]. Other articles stated their participants had received dietary restrictions without detailing the specific nutrition prescription, nor the counselling and compliance approaches used [48].

## Discussion

To the best of our knowledge, this is the first systematic review to quantitatively summarise the composition of the gut microbiota profile in adults with kidney disease or kidney stones compared to controls. There is consistent evidence that the gut microbial composition is altered in specific ways in adults with kidney disease and kidney stones. However, more research in this area is required to establish the specific role that these microbes have in kidney disease physiology and importantly, the clinical relevance in disease management. To further elucidate this, studies should employ more sophisticated microbial characterisation techniques appropriate for functional annotation (for instance shotgun sequencing) with the integration of other multi-omic technology such as metabolomics, as well as investigate the gut microbiota in larger sample sizes and different kidney disease populations. The findings from this review also highlighted a significant gap in the current evidence-base regarding a lack of reporting to control for the potential confounding effects of dietary intakes.

Lower bacterial diversity has been observed in a range of other clinical conditions, including inflammatory bowel disease (IBD), obesity, type 1 and 2 diabetes and coeliac disease [63, 64]. However, differences in the diversity of microbial communities between cohorts with kidney disease and kidney stones compared to controls remained inconclusive in this review. The overall dissimilarity in the microbial community structure as evaluated via β-diversity distance metrics was statistically significant in six studies. However, conflicting results were found for α-diversity measures. Seven studies provided evidence that α-diversity significantly reduced in adults with kidney disease or kidney stones relative to controls. In contrast, three studies reported an increase in microbial richness in adults with CKD, two of which investigated adults with ESKD [44, 55], including those receiving haemodialysis therapy [55]. It was inferred that the increase of microbial richness might reflect the proliferation of certain bacterial species [44, 55]. The overgrowth of microbes with pathogenic potential (pathobiont) has been observed along with increases in intestinal concentrations of uraemic toxins [65] associated with the progression of kidney disease, leading to the loss and breach in the intestinal epithelial barrier [65]. Dialysis is believed to worsen this epithelial barrier injury caused by CKD [66], partly due to intra-dialysis or post-dialysis hypotension bowel ischemia, and bowel oedema attributable to intra-dialysis fluid retention. Shi et al. [55] detected bacterial DNA in plasma samples of 27% of participants undergoing haemodialysis and 20% of their pre-dialysis CKD subjects. Interestingly, most of the bacterial DNA found in ESRD patients' blood was also found in their stool samples, but not in the dialysate solutions [55]. The researchers proposed that the bloodstream bacteria was primarily derived from the dysbiotic intestinal microbiota, and that HD exacerbates micro-inflammation in these patients to some degree by encouraging intestinal microbiota translocation due to an impaired intestinal barrier [55].

Changes at the phylum level with the elevation of Proteobacteria and decrease of Firmicutes was found in kidney disease cohorts. Previous studies have reported this enrichment of Proteobacteria is indicative of an unstable microbial structure [67] and has been correlated to diseases of inflammatory phenotype [67] such as cardiovascular disease and IBD [68]. Lipopolysaccharides (LPS) constitute the outer membranes of most Gram-negative bacteria [69], and bacterial members of Proteobacteria have been reported as potent LPS producers [70]. A connection between low-grade inflammation, sustained by LPS, and the development of metabolic disorders is well established, including evidence that indicates subclinical endotoxemia is a potential cause for inflammation in individuals with CKD [65]. A mechanistic exploration in male C57BL/6 mice showed that endotoxemia resulted in the activation of mTOR signalling in macrophages, leading to progressive kidney inflammatory injuries and subsequent fibrosis [71]. Several other taxa that were reportedly altered in adults with kidney disease have been linked to various clinical outcomes. For instance, *Streptococcus,* along with *Klebsiella* that was more abundant in kidney disease populations, has been positively associated with serum uraemic toxin TMAO levels [42]. Similarly, *Streptococcus* and *Blautia* were found to be related to other uraemic toxins such as IS and pCS and inversely associated with kidney function (eGFR) [62]. On the other hand, known commensal bacteria [9, 72] such *Prevotella, Roseburia* [51] and *Fecalibacterium prausnitzii* [47] that were depleted among kidney disease populations, were associated with a better kidney function (eGFR) [47, 51] and decreases in Cystatin C levels [51]. Similarly, *Prevotella, Prevotella 2, Prevotella 9 and Megamonas* were also associated with lower serum levels of IS and pCS [62], blood urea nitrogen (BUN) and creatinine [46, 62]. 

Findings related to the predicted biological functions of the microbial community in adults with kidney disease supports the notion that the gut microbiome may play an essential role in the production of ammonia from urea, and formation of uraemic toxin TMAO via the reduced decomposition of its precursor TMA. Genes relevant to choline, betaine, and L-carnitine metabolism were found to be downregulated [56], possibly resulting in the production of redundant TMA in the intestinal tract. In addition, the predicted expression of genes related to trimethylamine (TMA) metabolism were increased in adults with CKD [56]. Moreover, the majority of microbial families that were enriched in a cohort of participants receiving HD possessed the urease gene, while other highly abundant microbial families possessed the uricase gene, tryptophanase gene and p-cresol forming enzymes [36]. A reduction in bacterial families that possess the butyrate-kinase gene and phosphotransbutyrylase needed to produce butyrate, a four-carbon SCFA [36] was also identified among adults receiving HD therapy. The capacity to produce SCFAs is critical physiologically as they are needed to provide the energy for the growth and proliferation of colonocytes [73]; protection of the colonic epithelium from damage by reactive oxygen species and immune-modulating prostaglandins [73]; and aid in processes that reduce luminal pH associated with the inhibition of pathogenic microorganisms [74]. Interestingly, microbial genes essential to polyketide metabolism were also predicted to be significantly reduced in adults with CKD. Polyketides are a functionally diverse family of bioactive natural products and have many important uses for human health [75, 76]. For example, polyketides that are widely used include antibacterials (erythromycin), antifungals (amphotericin), anti-cancer agents (doxorubicin), immunosuppressants (rapamycin) and cholesterol-lowering agents [76].

Adults with kidney stones also had a unique gut microbiota profile compared to controls. For instance, *Bacteroides* were consistently reported to be in increased abundance, while *Dorea*, *Enterobacter*, *Christensenellaceae*, and its genus *Christensenellaceae R7 group,* decreased in adults with kidney stones*.* Discrepancies existed across studies concerning the up-regulation or down-regulation of microbial genes involved in oxalate degradation. Although in a small sample of kidney stone participants, shotgun sequencing analysis did indicate that the expression of genes involved in oxalate degradation were significantly reduced [50]. However, findings across included studies did agree that several microbes possessed functional oxalate-degrading properties, challenging the concept that the gut-nephrolithiasis-axis is merely limited to *Oxalobacter formigenes* [38, 50]. Other predicted functional activities of the gut microbiota involved in energy metabolism, glycan synthesis, lipid and carbohydrate metabolism were also altered compared to controls [38].

It is important to remark that predicting functional profiles through amplicon-based metagenomics (i.e. 16S rRNA sequencing), which was the most common approach employed by studies, offers only a limited resolution of the microbial communities’ functional potential and does not substitute shotgun sequencing [4]. However, the findings of both kidney disease and kidney stone populations uncovered through amplicon-based metagenomics do provide valuable functional insights for future research to undertake more in-depth explorations through shotgun metagenomics, metatranscriptomics and metaproteomics needed to further our understandings.

Overall, the application of other multi-omic technologies in gut microbiota-related investigations of adults with kidney disease or kidney stones was uncommon among the included studies. Although, some research groups did report results of their metabolomic analyses [39, 42, 56, 61, 62], a technique that involves identifying a set of metabolites within a sample [77]. For instance, De Angelis et al. [39] observed differences in faecal and urinary metabolome composition between IgAN patients and controls. The authors suggested that elevated serum-free amino acids detected in IgAN participants were possibly associated with lower absorption of gastrointestinal proteins, resulting in increased levels of faecal p-cresol due to enhanced microbial proteolytic metabolism and changes in the microbiota [39]. In a separately published paper with the same cohort already included in this review, Tao et al. [40] found that when using an untargeted metabolomics approach, individuals with DN could be distinguished from age and gender-matched diabetic controls by serum L-arginine (AUC = 0.824) and taurine (AUC = 0.789) levels. Nevertheless, greater employment of multi-omic technologies such as metabolomics needs to be integrated with comprehensive data on the functional capability of the microbiome and dietary intake in order to uncover some of the most challenging questions concerning the prevention and management of CKD and kidney disorders.

Modulation of the microbiome provides a new potential therapeutic target for preventing or personalising treatment in kidney disease and disorders. Despite hypotheses that postulate deleterious effects of specific diets and nutrients on the gastrointestinal microbiota regarding uraemic toxin generation in kidney disease [12, 26, 28, 78] and kidney stone formation [79], few explored this. Only three studies considered diet in their study design [42, 50, 61], of which only two provided adequate details of the dietary assessment methods used and considered diet as co-variates in the analysis of the microbiota. Further limitations within these studies existed. For instance, Barrios et al. [61] noted that information relating to dietary assessment and antibiotic use was only available for 11% of their sample, making interpretations of their results difficult. To date, the majority of the published studies have focused mainly on the effect of nutritional supplements such as prebiotics and probiotics to improve gut health and symptoms in individuals with CKD or kidney stones. Unfortunately, these types of therapies add further to the pill burden for this patient group [80] and have produced inconsistent results in either case [81–84]. Clinical trials that explore the effects of dietary components, such as dietary fibre in other clinical conditions are emerging [85]. Nevertheless, few studies have examined the effect of dietary patterns on the gut microbiome in CKD or kidney stones. Because nutrients are not consumed in isolation [86], exploring the impact of whole foods and overall dietary patterns on the gut microbiota may offer a superior and rigorous methodological approach.

Further research into the lifestyle and environmental exposures that differ across the spectrum of kidney disease conditions, and whether certain factors individually have a more significant impact, or whether there is a unifying effect on the microbiome, is essential to advance this area of research. For instance, along with subsequent dietary restrictions, other drug therapies and the dialysis procedure itself may explain variations observed between dialysis and non-dialysis CKD, but also between adults receiving PD compared to HD [1, 57]. For example, the clearance of metabolic wastes that may influence the microbiome differs as HD therapy is discontinuous, while PD works continuously [1]. Maio et al. [45] noted that there were significant differences in 58 bacterial taxa, seven of which decreased over 12 weeks following the use of phosphate binders (lanthanum carbonate). Hence, the impact of non-antibiotic drugs unique to people with CKD requires further exploration.

Our study has several limitations. Overall, the evaluation of results relating to alterations in the gut microbiota was challenging to evaluate mainly due to heterogeneity of the inclusion criteria of individuals recruited, methodologies used and reporting of results. It was thus not possible to conduct a meta-analysis. The majority of cited studies had small sample sizes and background information relating to the classification of controls, diet, comorbidities, medications and other lifestyle factors (such as smoking status, alcohol consumption and physical activity) were poorly accounted for— all of which may have influenced the results. The strength of this review is reflected in its systematic approach to highlight the existing state of evidence in the area of gut microbiota, kidney disease and kidney stone disease. It serves to unify methods and study designs needed to produce complementary findings and progress in this field of research.

## Conclusion

The gut microbiota profile of adults with kidney disease and kidney stones was consistently reported to be substantially different from controls. Evidence for altered genetic functions of the gut microbiota suggests a potential role of the gut microbiota in modulating host metabolism, particularly in the context of uraemic toxin generation in adults with kidney disease—Although greater investigation is still required. Studies with high statistical power, comparable and reproducible methods that include validated dietary assessment, as well as the combined utilisation of more sophisticated multi-omic technologies, are required to map functional capabilities and more clearly elucidate the role of the microbiota in kidney health.

## Supplementary information

**Additional file 1.** Supplementary Data

## Data Availability

The datasets supporting the conclusions of this article are included within the article and/or its additional files. Please refer to the manuscript’s supplementary information (Additional file 1) for the following information: − Final search strategy for all databases can be found in *Item 1.* − Datasets presenting the reported direction of microbial alteration in adults with kidney disease can be found in *Table S1.* − Datasets presenting the reported direction of microbial alteration in adults with kidney stones can be found in *Table S2.* − Presence and absence of gene bearing species studied by the conserved genes such as 16S rRNA gene (archaea), 18S rRNA gene (microeukaryotes) and ITS region (fungi) sequencing results which has been summarised from Suryavanshi et al. 2018 [37] can be found in *Table S3.*

## Abbreviations

AUC: Area under the ROC curve
BUN: Blood urea nitrogen
CKD: Chronic kidney disease
DGGE: Denaturing gradient gel electrophoresis
DN: Diabetic nephropathy
DNA: Deoxyribonucleic acid
eGFR: Estimated glomerular filtration rate
EPIC FFQ: European Prospective Investigation of Cancer and Nutrition Food Frequency Questionnaire
ESKD: End-stage kidney disease
FFQ: Food Frequency Questionnaire
FISH: Fluorescence in situ hybridization
HD: Haemodialysis
IBD: Inflammatory bowel disease
IgAN: IgA nephropathy
IS: Indoxyl sulphate
ITS: Internal transcribed spacer
KEGG database: Kyoto Encyclopedia of Genes and Genomes database
KT: Kidney Transplant
LPS: Lipopolysaccharides
NOS: Newcastle-Ottawa Scale
PAG: Phenylacetylglutamine
PCR: Polymerase chain reaction
pCS: p-Cresyl sulphate
PD: Peritoneal dialysis
PICRUSt: Phylogenetic Investigation of Communities by Reconstruction of Unobserved States
PRISMA: Preferred Reporting Items for Systematic Reviews and Meta-Analyses
qPCR: quantitative real-time polymerase chain reaction
ROC curve: Receiver operating characteristic curve
rRNA: Ribosomal ribonucleic acid
SCFA: Short-chain fatty acids
T2DM: Type 2 Diabetes Mellitus
TMA: Trimethylamine
TMAO: Trimethylamine N-Oxide
