# Supplementary material for: The gut microbiota profile of adults with kidney disease and kidney stones: a systematic review of the literature
Source: BMC Nephrol. 2020 Jun 5;21:215. doi: 10.1186/s12882-020-01805-w (PMC7275316; doi:10.1186/s12882-020-01805-w)
Supplement: Supplementary file 1 — Additional file 1. Supplementary Data [file 12882_2020_1805_MOESM1_ESM.docx]

**SUPPLEMENTARY DATA**

**The gut microbiota profile of adults with kidney disease and kidney disorders: a systematic review of the literature**

Jordan Stanford^1-2^, Karen Charlton^1-3^, Anita Stefoska-Needham^1-3^, Rukayat Ibrahim^4^, Kelly Lambert^1-3^

**Item 1:** Complete database search strategy – pages 2-8

**Table S1**: Dataset for kidney diseases – pages 9-12

**Table S2:** Dataset for kidney stones – pages 13-15

**Table S3.** Presence and absence of gene bearing species studied by the conserved genes such as 16S rRNA gene (archaea), 18S rRNA gene (microeukaryotes) and ITS region (fungi) sequencing results which has been summarised from Suryavanshi et al. 2018 – page 16

**Item 1:** Complete database search strategy.

**SCIENTIFIC DATABASES**

| **Database/platform:** | **CINAHL** |
| --- | --- |
| **Library:** | University of Wollongong |
| **Date of search** | 13/07/2018 |
| **Comments:** | Expanders- “apply to equivalent subjects” applied |
| **Search query:** | (("Chronic kidney disease*") OR (MH "Kidney Failure, Chronic") OR ("end stage renal failure") OR ("end stage renal disease") OR ("end stage kidney disease") OR ("renal disease*") OR ("renal failure") OR ("kidney insufficienc*") OR ("renal insufficienc*") OR (MH "Renal Insufficiency") OR (MH "Renal Insufficiency, Chronic") OR ("Hemodialysis") OR ("Peritoneal dialysis") OR (MH "peritoneal dialysis ") OR (MH "renal dialysis") OR (MH "dialysis") OR ("dialysis") OR ("Kidney transplant*") OR (MH "kidney transplantation") OR ("Kidney disease*") OR (MH "Kidney Diseases") OR ("Kidney failure") OR ("Kidney stone*") OR ("renal calculi") OR ("renal calculus") OR (MH "Kidney Calculi") OR ("nephrolithiasis") OR ("Glomerulonephritis") OR (MH "Glomerulonephritis") OR ("Nephrotic syndrome") OR (MH "Nephrotic Syndrome") OR ("Polycystic kidney disease") OR (MH "Polycystic Kidney Diseases") OR ("Alport syndrome") OR (MH "Nephritis, Hereditary") OR ("Fabry disease") OR (MH "Fabry Disease") OR ("kidney") OR ("renal") OR (MH “Renal Replacement Therapy”) OR (“Renal Replacement Therap*”))  AND  (("Gut Health") OR ("Gut flora") OR ("Gut microflora") OR ("Gut microbio*") OR (MH "gastrointestinal microbiome") OR ("Gastrointestinal microbio*") OR ("Gastrointestinal Health") OR ("Gastrointestinal flora") OR ("Gastrointestinal microflora") OR ("intestinal microbio*") OR ("intestinal Health") OR ("intestinal flora") OR ("intestinal microflora") OR ("Enteric bacteria")OR (MH "Microbiota")) |
| **Number of hits** | 109 hits (original search performed August 7^th^ 2018)  55 new hits (updated search performed October 3^rd^ 2019); applied since 2018 filter |
| **Notes** |  |

| **Database/platform:** | **Cochrane Library** |  |
| --- | --- | --- |
| **Library:** | University of Wollongong |  |
| **Date of search** | 13/07/2018 |  |
| **Limits:** | None applied |  |
| **Search query:** |  |  |
| #1 | ("Chronic kidney disease*" or "end stage renal failure" or "end stage renal disease" or "end stage kidney disease" or "renal disease*" or "renal failure" or "kidney insufficienc*" or "renal insufficienc*" or "Hemodialysis" or "Peritoneal dialysis" or "dialysis" or "Kidney transplant*" or "Kidney disease*" or "Kidney failure" or "Kidney stone*" or "renal calculi" or "renal calculus" or "nephrolithiasis" or "Glomerulonephritis" or "Nephrotic syndrome" or "Polycystic kidney disease" or "Alport syndrome" or "Fabry disease" or "kidney" or "renal" or "Renal Replacement Therap*") | |
| #2 | ("Gut Health" or "Gut flora" or "Gut microflora" or "Gut microbio*" or "Gastrointestinal microbio*" or "Gastrointestinal Health" or "Gastrointestinal flora" or "Gastrointestinal microflora" or "intestinal microbio*" or "intestinal Health" or "intestinal flora" or "intestinal microflora" or "Enteric bacteria") | |
| #3 | MeSH descriptor: [Kidney Diseases] this term only | |
| #4 | MeSH descriptor: [Kidney Failure, Chronic] this term only | |
| #5 | MeSH descriptor: [Renal Insufficiency] this term only | |
| #6 | MeSH descriptor: [Renal Insufficiency, Chronic] this term only | |
| #7 | MeSH descriptor: [Renal Dialysis] this term only | |
| #8 | MeSH descriptor: [Dialysis] this term only | |
| #9 | MeSH descriptor: [Peritoneal Dialysis] this term only | |
| #10 | MeSH descriptor: [Kidney Transplantation] this term only | |
| #11 | MeSH descriptor: [Kidney Calculi] this term only | |
| #12 | MeSH descriptor: [Glomerulonephritis] this term only | |
| #13 | MeSH descriptor: [Nephrotic Syndrome] this term only | |
| #14 | MeSH descriptor: [Polycystic Kidney Diseases] this term only | |
| #15 | MeSH descriptor: [Nephritis, Hereditary] this term only | |
| #16 | MeSH descriptor: [Fabry Disease] this term only | |
| #17 | MeSH descriptor: [Renal Replacement Therapy] this term only | |
| #18 | MeSH descriptor: [Gastrointestinal Microbiome] this term only | |
| #19 | MeSH descriptor: [Microbiota] this term only | |
| #20 | #1 or #3 or #4 or #5 or #6 or #7 or #8 or #9 or #10 or #11 or #12 or #13 or #14 or #15 or #16 or #17 | |
| #21 | #2 or #18 or #19 | |
| #22 | #20 and #21 | |
| **Number of hits** | 90 hits (original search performed August 7^th^ 2018)  6 new hits (updated search performed October 3^rd^ 2019) | |
| **Notes** |  | |

| **Database/platform:** | **MEDLINE** |
| --- | --- |
| **Library:** | Free access |
| **Date of search** | 13/07/2018 |
| **Limits:** | None applied |
| **Search query:** | (("Chronic kidney disease*") OR (MH "Kidney Failure, Chronic") OR ("end stage renal failure") OR ("end stage renal disease") OR ("end stage kidney disease") OR ("renal disease*") OR ("renal failure") OR ("kidney insufficienc*") OR ("renal insufficienc*") OR (MH "Renal Insufficiency") OR (MH "Renal Insufficiency, Chronic") OR ("Hemodialysis") OR ("Peritoneal dialysis") OR (MH "peritoneal dialysis ") OR (MH "renal dialysis") OR (MH "dialysis") OR ("dialysis") OR ("Kidney transplant*") OR (MH "kidney transplantation") OR ("Kidney disease*") OR (MH "Kidney Diseases") OR ("Kidney failure") OR ("Kidney stone*") OR ("renal calculi") OR ("renal calculus") OR (MH "Kidney Calculi") OR ("nephrolithiasis") OR ("Glomerulonephritis") OR (MH "Glomerulonephritis") OR ("Nephrotic syndrome") OR (MH "Nephrotic Syndrome") OR ("Polycystic kidney disease") OR (MH "Polycystic Kidney Diseases") OR ("Alport syndrome") OR (MH "Nephritis, Hereditary") OR ("Fabry disease") OR (MH "Fabry Disease") OR ("kidney") OR ("renal") OR (MH “Renal Replacement Therapy”))  AND (("Gut Health") OR ("Gut flora") OR ("Gut microflora") OR ("Gut microbio*") OR (MH "gastrointestinal microbiome") OR ("Gastrointestinal microbio*") OR ("Gastrointestinal Health") OR ("Gastrointestinal flora") OR ("Gastrointestinal microflora") OR ("intestinal microbio*") OR ("intestinal Health") OR ("intestinal flora") OR ("intestinal microflora") OR ("Enteric bacteria")OR (MH "Microbiota")) |
| **Number of hits** | 907 hits (original search performed August 7^th^ 2018)  249 new hits (updated search performed October 3^rd^ 2019) |
| **Notes** |  |

| **Database/platform:** | **PubMed** |
| --- | --- |
| **Library:** | Free access |
| **Date of search** | 13/07/2018 |
| **Limits:** | None applied |
| **Search query:** | ("Chronic kidney disease*"[All Fields] OR "end stage kidney disease"[All Fields] OR "kidney failure, chronic"[MeSH Terms] OR "end stage renal failure"[All Fields] OR "end stage renal disease"[All Fields] OR "renal disease*"[All Fields] OR "renal failure"[All Fields] OR "kidney insufficiency"[All Fields] OR "kidney insufficiencies"[All Fields] OR "renal insufficiency"[All Fields] OR "renal insufficiencies"[All Fields] OR "renal insufficiency, chronic"[MeSH Terms] OR "renal insufficiency"[MeSH Terms] OR "Hemodialysis"[All Fields] OR "Peritoneal dialysis"[All Fields] OR "peritoneal dialysis"[MeSH Terms] OR "renal dialysis"[MeSH Terms] OR "dialysis"[All Fields] OR "dialysis"[MeSH Terms] OR "Kidney transplant*"[All Fields] OR "kidney transplantation"[MeSH Terms] OR "Kidney disease*"[All Fields] OR "Kidney Diseases"[MeSH Terms] OR "Kidney failure"[All Fields] OR "Kidney stone*"[All Fields] OR "renal calculi"[All Fields] OR "renal calculus"[All Fields] OR "Kidney Calculi"[MeSH Terms] OR "nephrolithiasis"[All Fields] OR "Glomerulonephritis"[All Fields] OR "Glomerulonephritis"[MeSH Terms] OR "Nephrotic syndrome"[All Fields] OR "Nephrotic Syndrome"[MeSH Terms] OR "Polycystic kidney disease"[All Fields] OR "Polycystic Kidney Diseases"[MeSH Terms] OR "Alport syndrome"[All Fields] OR "Nephritis, Hereditary"[MeSH Terms] OR "Fabry disease"[All Fields] OR "Fabry Disease"[MeSH Terms] OR "kidney"[All Fields] OR "renal"[All Fields] OR "Renal Replacement Therapy"[MeSH Terms]) AND ("Gut Health"[All Fields] OR "Gut flora"[All Fields] OR "Gut microflora"[All Fields] OR "Gut microbiome"[All Fields] OR "Gut microbiota"[All Fields] OR "gastrointestinal microbiome"[MeSH Terms] OR "Gastrointestinal microbiome"[All Fields] OR "Gastrointestinal microbiota"[All Fields] OR "Gastrointestinal Health"[All Fields] OR "Gastrointestinal flora"[All Fields] OR "Gastrointestinal microflora"[All Fields] OR "intestinal microbiome"[All Fields] OR "intestinal microbiota"[All Fields] OR "intestinal Health"[All Fields] OR "intestinal flora"[All Fields] OR "intestinal microflora"[All Fields] OR "Enteric bacteria"[All Fields] OR "Microbiota"[MeSH Terms]) |
| **Number of hits** | 833 hits (original search performed August 7^th^ 2018)  320 new hits (updated search performed October 3^rd^ 2019) |
| **Notes** |  |

| **Database/platform:** | **Web of Science** |
| --- | --- |
| **Library:** | University of Wollongong |
| **Date of search** | 13/07/2018 |
| **Limits:** | None applied |
| **Search query:** | (( "Chronic kidney disease*" OR "end stage renal failure" OR "end stage renal disease" OR "end stage kidney disease" OR "renal disease*" OR "renal failure" OR "kidney insufficienc*" OR "renal insufficienc*" OR "Hemodialysis" OR "Peritoneal dialysis" OR "dialysis" OR "Kidney transplant*" OR "Kidney disease*" OR "Kidney failure" OR "Kidney stone*" OR "renal calculi" OR "renal calculus" OR "nephrolithiasis" OR "Glomerulonephritis" OR "Nephrotic syndrome" OR "Polycystic kidney disease" OR "Alport syndrome" OR "Fabry disease" OR "kidney" OR "renal" OR "Renal Replacement Therap*" )) AND TOPIC: (( "Gut Health" OR "Gut flora" OR "Gut microflora" OR "Gut microbio*" OR "Gastrointestinal microbio*" OR "Gastrointestinal Health" OR "Gastrointestinal flora" OR "Gastrointestinal microflora" OR "intestinal microbio*" OR "intestinal Health" OR "intestinal flora" OR "intestinal microflora" OR "Enteric bacteria" )) |
| **Number of hits** | 842 hits (original search performed August 7^th^ 2018)  415 new hits (updated search performed October 3^rd^ 2019) |
| **Notes** |  |

| **Database/platform:** | **Scopus** |
| --- | --- |
| **Library:** | University of Wollongong |
| **Date of search** | 13/07/2018 |
| **Limits:** | None applied |
| **Search query:** | TITLE-ABS-KEY ( ( "Chronic kidney disease*" OR "end stage renal failure" OR "end stage renal disease" OR "end stage kidney disease" OR "renal disease*" OR "renal failure" OR "kidney insufficienc*" OR "renal insufficienc*" OR "Hemodialysis" OR "Peritoneal dialysis" OR "dialysis" OR "Kidney transplant*" OR "Kidney disease*" OR "Kidney failure" OR "Kidney stone*" OR "renal calculi" OR "renal calculus" OR "nephrolithiasis" OR "Glomerulonephritis" OR "Nephrotic syndrome" OR "Polycystic kidney disease" OR "Alport syndrome" OR "Fabry disease" OR "kidney" OR "renal" OR "Renal Replacement Therap*" ) ) AND TITLE-ABS-KEY ( ( "Gut Health" OR "Gut flora" OR "Gut microflora" OR "Gut microbio*" OR "Gastrointestinal microbio*" OR "Gastrointestinal Health" OR "Gastrointestinal flora" OR "Gastrointestinal microflora" OR "intestinal microbio*" OR "intestinal Health" OR "intestinal flora" OR "intestinal microflora" OR "Enteric bacteria" ) ) |
| **Number of hits** | 938 hits (original search performed August 7^th^ 2018)  243 new hits (updated search performed October 3^rd^ 2019) |
| **Notes** |  |

**GREY LITERATURE DATABASES**

| **Database/platform:** | **Google scholar** |
| --- | --- |
| **Date of search** | 18/07/2018, 2:51PM |
| **Limits** | None applied |
| **Search query** | (microbiome OR microbiota OR "intestinal flora") AND (kidney OR “kidney disease” OR renal OR dialysis OR "renal replacement" OR "end stage kidney disease" OR "end stage renal disease" OR "kidney stone" OR nephrolithiasis OR Glomerulonephritis OR "Nephrotic syndrome") |
| **Number of hits** | Screened first 100 hits, all 100 potentially relevant (sorted by relevance) |
| **Notes** | Limited capacity of words, unable to add PKD, Alport syndrome & Fabrys disease into search terms (due to length of space).  Repeated Google Scholar search 3^rd^ October 2019- imported first 100 hits. |

| **Database/platform:** | **TROVE** |
| --- | --- |
| **Date of search** | 18/07/2018, 11:42 AM |
| **Limits** | None applied |
| **Search query** | title:(microbiota OR microbiome OR "gut flora" OR "microflora" OR "gastrointestinal flora" OR "gastrointestinal microflora" OR "intestinal flora" OR "intestinal microflora" OR "enteric bacteria") AND (kidney OR renal OR dialysis OR "renal replacement" OR "end stage kidney disease" OR "end stage renal disease" OR "kidney stone" OR nephrolithiasis OR Glomerulonephritis OR "Nephrotic syndrome") |
| **Number of hits** | 391 |
| **Notes** | Screened first 100 hits (ordered by relevance) |

| **Database/platform:** | **National Kidney Foundation** |
| --- | --- |
| **Date of search** | 18/07/2018, 09:23 AM |
| **Limits** | None applied |
| **Search query** | Gut microbiome – **18 hits**  Microbiome- **4 hits**  Microbiota- **1 hit**  Kidney AND gut Microbiome- **12 028 hits** |
| **Number of hits** | Screened first 100 hits- 0 relevant |
| **Notes** | Screened first 100 hits- 0 relevant |

| **Database/platform:** | **Google.com** |
| --- | --- |
| **Date of search** | 18/07/2018. 5:37pm |
| **Limits** | None applied |
| **Search query** | (microbiome OR microbiota OR "intestinal flora") AND (kidney OR “kidney disease” OR renal OR dialysis OR "renal replacement" OR "end stage kidney disease" OR "end stage renal disease" OR "kidney stone" OR nephrolithiasis OR Glomerulonephritis OR "Nephrotic syndrome") - **2,890,000 hits** |
| **Number of hits** | 78 relevant |
| **Notes** | Screened first 100 hits, utilised advanced google search tool. |

| **Database/platform:** | **The Joanna Briggs Institute** |
| --- | --- |
| **Date of search** | 19/07/2018 1:31PM |
| **Limits** | None applied |
| **Search query** | Kidney AND microbiome – 0 hits  Kidney**- 64 hits** |
| **Number of hits** | 0 relevant |
| **Notes** |  |

| **Database/platform:** | **NICE** |
| --- | --- |
| **Date of search** | 19/07/2018 1:38PM |
| **Limits** | None applied |
| **Search query** | (microbiome OR microbiota OR "intestinal flora") AND (kidney OR renal OR dialysis OR "renal replacement" OR "end stage kidney disease" OR "end stage renal disease" OR "kidney stone" OR nephrolithiasis OR Glomerulonephritis OR "Nephrotic syndrome")**- 168 hits** |
| **Number of hits** | 4 relevant |
| **Notes** | Screened first 100 results |

| **Database/platform:** | **TRIP** |
| --- | --- |
| **Date of search** | 19/07/2018 2:32PM |
| **Limits** | None applied |
| **Search query** | (microbiota OR microbiome OR "gut flora" OR "microflora" OR "gastrointestinal flora" OR "gastrointestinal microflora" OR "intestinal flora" OR "intestinal microflora" OR "enteric bacteria") AND (kidney OR renal OR dialysis OR "renal replacement" OR "end stage kidney disease" OR "end stage renal disease" OR "kidney stone" OR nephrolithiasis OR Glomerulonephritis OR "Nephrotic syndrome")**- 914 hits** |
| **Number of hits** | 31 relevant |
| **Notes** | Screened first 100, sorted by relevance;  Alternatives trailed: ** with PKD, Alport, fabrys- got 915 hits, |

**Table S1.** Dataset presenting the reported direction of microbial alteration in adults with kidney disease compared to controls.

| **Taxonomy** | **Microbiota** | **Total** | **Decreased findings** | | | | **Increased findings** | | | | **Level of evidence*** |
| --- | --- | --- | --- | --- | --- | --- | --- | --- | --- | --- | --- |
|  |  | Total no. studies reporting these taxa | No. Studies reporting decreased findings | Consistent percentage | Decreased findings (good quality) | Decreased findings (poor quality) | No. Studies reporting increased findings | Consistent percentage | Increased findings (good quality) | Increased findings (poor quality) | Rating |
| Phylum | Deferribacteres | 1 | 1 | 100% | 1 | 0 | 0 | 0% | 0 | 0 | Weak |
| Phylum | Firmicutes | 5 | 4 | 80% | 1 | 3 | 1 | 20% | 1 | 0 | Strong |
| Phylum | Fusobacteria | 1 | 0 | 0% | 0 | 0 | 1 | 100% | 1 | 0 | Weak |
| Phylum | Proteobacteria | 5 | 0 | 0% | 0 | 0 | 5 | 100% | 2 | 3 | Strong |
| Phylum | Synergistetes | 1 | 1 | 100% | 1 | 0 | 0 | 0% | 0 | 0 | Weak |
| Class | Actinobacteria | 2 | 0 | 0% | 0 | 0 | 2 | 100% | 2 | 0 | Strong |
| Class | Alphaproteobacteria | 3 | 0 | 0% | 0 | 0 | 3 | 100% | 2 | 1 | Strong |
| Class | Betaproteobacteria | 1 | 1 | 100% | 1 | 0 | 0 | 0% | 0 | 0 | Weak |
| Class | Coriobacteriia | 1 | 0 | 0% | 0 | 0 | 1 | 100% | 1 | 0 | Weak |
| Class | Fusobacteriia | 1 | 0 | 0% | 0 | 0 | 1 | 100% | 1 | 0 | Weak |
| Class | Gammaproteobacteria | 4 | 0 | 0% | 0 | 0 | 4 | 100% | 2 | 2 | Strong |
| Class | Synergistia | 1 | 1 | 100% | 1 | 0 | 0 | 0% | 0 | 0 | Weak |
| Order | Bifidobacteriales | 1 | 0 | 0% | 0 | 0 | 1 | 100% | 1 | 0 | Weak |
| Order | Campylobacterales | 1 | 1 | 100% | 1 | 0 | 0 | 0% | 0 | 0 | Weak |
| Order | Coriobacteriales | 3 | 0 | 0% | 0 | 0 | 3 | 100% | 3 | 0 | Strong |
| Order | Deferribacterales | 1 | 1 | 100% | 1 | 0 | 0 | 0% | 0 | 0 | Weak |
| Order | Enterobacteriales | 4 | 1 | 25% | 1 | 0 | 3 | 75% | 1 | 2 | Moderate |
| Order | Erysipelotrichales | 1 | 0 | 0% | 0 | 0 | 1 | 100% | 1 | 0 | Weak |
| Order | Fusobacteriales | 1 | 0 | 0% | 0 | 0 | 1 | 100% | 1 | 0 | Weak |
| Order | Neisseriales | 1 | 0 | 0% | 0 | 0 | 1 | 100% | 1 | 0 | Weak |
| Order | Rhizobiales | 1 | 1 | 100% | 1 | 0 | 0 | 0% | 0 | 0 | Weak |
| Order | Rickettisales | 1 | 1 | 100% | 1 | 0 | 0 | 0% | 0 | 0 | Weak |
| Order | Synergistales | 1 | 1 | 100% | 1 | 0 | 0 | 0% | 0 | 0 | Weak |
| Family | 0319_6G20 | 1 | 1 | 100% | 1 | 0 | 0 | 0% | 0 | 0 | Weak |
| Family | Acidaminococcaceae | 1 | 0 | 0% | 0 | 0 | 1 | 100% | 1 | 0 | Weak |
| Family | Aeromonadaceae | 1 | 1 | 100% | 1 | 0 | 0 | 0% | 0 | 0 | Weak |
| Family | Alcaligenaceae | 4 | 3 | 75% | 1 | 2 | 1 | 25% | 1 | 0 | Moderate |
| Family | Anaplasmataceae | 1 | 1 | 100% | 1 | 0 | 0 | 0% | 0 | 0 | Weak |
| Family | Bacteroidales family S24-7 | 2 | 2 | 100% | 2 | 0 | 0 | 0% | 0 | 0 | Strong |
| Family | Bradyrhizobiaceae | 1 | 1 | 100% | 1 | 0 | 0 | 0% | 0 | 0 | Weak |
| Family | Campylobacteraceae | 1 | 0 | 0% | 0 | 0 | 1 | 100% | 1 | 0 | Weak |
| Family | Christensenellaceae | 1 | 1 | 100% | 1 | 0 | 0 | 0% | 0 | 0 | Weak |
| Family | Christensenellaceae R-7 Group | 1 | 1 | 100% | 1 | 0 | 0 | 0% | 0 | 0 | Weak |
| Family | Clostridiaceae 1 | 1 | 0 | 0% | 0 | 0 | 1 | 100% | 1 | 0 | Weak |
| Family | Colwellaceae | 1 | 1 | 100% | 1 | 0 | 0 | 0% | 0 | 0 | Weak |
| Family | Comamonadaceae | 1 | 0 | 0% | 0 | 0 | 1 | 100% | 1 | 0 | Weak |
| Family | Coriobacteriaceae | 4 | 0 | 0% | 0 | 0 | 4 | 100% | 3 | 1 | Strong |
| Family | Corynebacteriaceae | 1 | 0 | 0% | 0 | 0 | 1 | 100% | 1 | 0 | Weak |
| Family | Deferribacteraceae | 1 | 1 | 100% | 1 | 0 | 0 | 0% | 0 | 0 | Weak |
| Family | Enterobacteriaceae | 7 | 1 | 14% | 1 | 0 | 6 | 86% | 2 | 4 | Strong |
| Family | Enterococcaceae | 3 | 0 | 0% | 0 | 0 | 3 | 100% | 2 | 1 | Strong |
| Family | Fusobacteriaceae | 1 | 0 | 0% | 0 | 0 | 1 | 100% | 1 | 0 | Weak |
| Family | Helicobacteraceae | 1 | 1 | 100% | 1 | 0 | 0 | 0% | 0 | 0 | Weak |
| Family | Intrasporangiaceae | 1 | 1 | 100% | 1 | 0 | 0 | 0% | 0 | 0 | Weak |
| Family | Methylobacteriaceae | 1 | 0 | 0% | 0 | 0 | 1 | 100% | 1 | 0 | Weak |
| Family | Mycobacteriaceae | 1 | 1 | 100% | 1 | 0 | 0 | 0% | 0 | 0 | Weak |
| Family | Neisseriaceae | 1 | 0 | 0% | 0 | 0 | 1 | 100% | 1 | 0 | Weak |
| Family | Prevotellaceae | 6 | 6 | 100% | 2 | 4 | 0 | 0% | 0 | 0 | Strong |
| Family | Rhodospirillaceae | 1 | 0 | 0% | 0 | 0 | 1 | 100% | 1 | 0 | Weak |
| Family | Rikenellaceae | 2 | 2 | 100% | 2 | 0 | 0 | 0% | 0 | 0 | Strong |
| Family | Streptococcaceae | 3 | 0 | 0% | 0 | 0 | 3 | 100% | 2 | 1 | Strong |
| Family | Sutterellaceae | 1 | 1 | 100% | 1 | 0 | 0 | 0% | 0 | 0 | Weak |
| Family | Synergistaceae | 1 | 1 | 100% | 1 | 0 | 0 | 0% | 0 | 0 | Weak |
| Family | Veillonellaceae | 2 | 2 | 100% | 1 | 1 | 0 | 0% | 0 | 0 | Moderate |
| Genus | Acidaminococcus | 1 | 1 | 100% | 1 | 0 | 0 | 0% | 0 | 0 | Weak |
| Genus | Acidovorax | 1 | 1 | 100% | 1 | 0 | 0 | 0% | 0 | 0 | Weak |
| Genus | Agromyces | 1 | 0 | 0% | 0 | 0 | 1 | 100% | 1 | 0 | Weak |
| Genus | Alistipes | 1 | 1 | 100% | 1 | 0 | 0 | 0% | 0 | 0 | Weak |
| Genus | Allobaculum | 1 | 1 | 100% | 1 | 0 | 0 | 0% | 0 | 0 | Weak |
| Genus | Alloprevotella | 1 | 0 | 0% | 0 | 0 | 1 | 100% | 1 | 0 | Weak |
| Genus | Asteroleplasma | 1 | 1 | 100% | 1 | 0 | 0 | 0% | 0 | 0 | Weak |
| Genus | Asticcacaulis | 1 | 0 | 0% | 0 | 0 | 1 | 100% | 1 | 0 | Weak |
| Genus | Atopobium | 1 | 1 | 100% | 1 | 0 | 0 | 0% | 0 | 0 | Weak |
| Genus | Bacteroides | 4 | 1 | 25% | 1 | 0 | 3 | 75% | 0 | 3 | Weak |
| Genus | Bifidobacterium | 4 | 3 | 75% | 0 | 3 | 1 | 25% | 1 | 0 | Weak |
| Genus | Bilophila | 2 | 0 | 0% | 0 | 0 | 2 | 100% | 1 | 1 | Moderate |
| Genus | Blautia | 2 | 0 | 0% | 0 | 0 | 2 | 100% | 2 | 0 | Strong |
| Genus | Caproiciproducens | 1 | 0 | 0% | 0 | 0 | 1 | 100% | 1 | 0 | Weak |
| Genus | Clostridium IV | 1 | 0 | 0% | 0 | 0 | 1 | 100% | 1 | 0 | Weak |
| Genus | Clostridium sensu stricto | 1 | 0 | 0% | 0 | 0 | 1 | 100% | 1 | 0 | Weak |
| Genus | Collimonas | 1 | 1 | 100% | 1 | 0 | 0 | 0% | 0 | 0 | Weak |
| Genus | Coprococcus | 3 | 3 | 100% | 1 | 2 | 0 | 0% | 0 | 0 | Moderate |
| Genus | Desulfosporosinus | 1 | 1 | 100% | 1 | 0 | 0 | 0% | 0 | 0 | Weak |
| Genus | Desulfotomaculum | 1 | 0 | 0% | 0 | 0 | 1 | 100% | 1 | 0 | Weak |
| Genus | Desulfovibrio | 3 | 0 | 0% | 0 | 0 | 3 | 100% | 1 | 2 | Moderate |
| Genus | Eisenbergiella | 1 | 0 | 0% | 0 | 0 | 1 | 100% | 1 | 0 | Weak |
| Genus | Enterorhabdus | 2 | 2 | 100% | 2 | 0 | 0 | 0% | 0 | 0 | Strong |
| Genus | Erysipelatoclostridium | 2 | 0 | 0% | 0 | 0 | 2 | 100% | 1 | 1 | Moderate |
| Genus | Erysipelotrichaceae UCG-003 | 1 | 0 | 0% | 0 | 0 | 1 | 100% | 1 | 0 | Weak |
| Genus | Erysipelotrichaceae UCG-006 | 1 | 0 | 0% | 0 | 0 | 1 | 100% | 1 | 0 | Weak |
| Genus | Escherichia-Shigella | 4 | 1 | 25% | 1 | 0 | 3 | 75% | 1 | 1 | Moderate |
| Genus | Eubacterium | 1 | 1 | 100% | 1 | 0 | 0 | 0% | 0 | 0 | Weak |
| Genus | Eubacterium rectale group | 1 | 0 | 0% | 0 | 0 | 1 | 100% | 1 | 0 | Weak |
| Genus | Eubacterium ventriosum group | 1 | 1 | 100% | 1 | 0 | 0 | 0% | 0 | 0 | Weak |
| Genus | Eubacterium ventriosum group | 1 | 1 | 100% | 1 | 0 | 0 | 0% | 0 | 0 | Weak |
| Genus | Faecalibacterium | 3 | 3 | 100% | 1 | 2 | 0 | 0% | 0 | 0 | Moderate |
| Genus | Faecalitalea | 1 | 0 | 0% | 0 | 0 | 1 | 100% | 1 | 0 | Weak |
| Genus | Family XIII AD3011 group | 1 | 0 | 0% | 0 | 0 | 1 | 100% | 1 | 1 | Weak |
| Genus | Flavobacterium | 1 | 1 | 100% | 1 | 0 | 0 | 0% | 0 | 0 | Weak |
| Genus | Fusobacterium | 1 | 0 | 0% | 0 | 0 | 1 | 100% | 1 | 0 | Weak |
| Genus | Haemophilus | 1 | 0 | 0% | 0 | 0 | 1 | 100% | 1 | 0 | Weak |
| Genus | Helicobacter | 1 | 1 | 100% | 1 | 0 | 0 | 0% | 0 | 0 | Weak |
| Genus | Herbaspirillum | 1 | 0 | 0% | 0 | 0 | 1 | 100% | 1 | 0 | Weak |
| Genus | Hungatella | 1 | 0 | 0% | 0 | 0 | 1 | 100% | 1 | 0 | Weak |
| Genus | Klebsiella | 3 | 0 | 0% | 0 | 0 | 3 | 100% | 1 | 2 | Moderate |
| Genus | Knoellia | 1 | 1 | 100% | 1 | 0 | 0 | 0% | 0 | 0 | Weak |
| Genus | Lachnoclostridium | 1 | 0 | 0% | 0 | 0 | 1 | 100% | 1 | 0 | Weak |
| Genus | Lachnospiraceae UCG-004 | 1 | 1 | 100% | 1 | 0 | 0 | 0% | 0 | 0 | Weak |
| Genus | Lactococcus | 1 | 1 | 100% | 1 | 0 | 0 | 0% | 0 | 0 | Weak |
| Genus | Leuconostoc | 1 | 1 | 100% | 1 | 0 | 0 | 0% | 0 | 0 | Weak |
| Genus | Megamonas | 2 | 2 | 100% | 2 | 0 | 0 | 0% | 0 | 0 | Strong |
| Genus | Methylobacterium | 1 | 0 | 0% | 0 | 0 | 1 | 100% | 1 | 0 | Weak |
| Genus | Mucispirlium | 1 | 1 | 100% | 1 | 0 | 0 | 0% | 0 | 0 | Weak |
| Genus | Mycobacterium | 1 | 1 | 100% | 1 | 0 | 0 | 0% | 0 | 0 | Weak |
| Genus | Neisseria | 1 | 0 | 0% | 0 | 0 | 1 | 100% | 1 | 0 | Weak |
| Genus | Niabella | 1 | 0 | 0% | 0 | 0 | 1 | 100% | 1 | 0 | Weak |
| Genus | Olsenella | 1 | 1 | 100% | 1 | 0 | 0 | 0% | 0 | 0 | Weak |
| Genus | Oribacterium | 2 | 2 | 100% | 1 | 1 | 0 | 0% | 0 | 0 | Moderate |
| Genus | Parabacteroides | 4 | 1 | 25% | 1 | 0 | 3 | 75% | 0 | 3 | Weak |
| Genus | Parvibacter | 1 | 1 | 100% | 1 | 0 | 0 | 0% | 0 | 0 | Weak |
| Genus | Pelomonas | 1 | 1 | 100% | 1 | 0 | 0 | 0% | 0 | 0 | Weak |
| Genus | Phascolarctobacterium | 2 | 0 | 0% | 0 | 0 | 2 | 100% | 1 | 1 | Moderate |
| Genus | Prevotella | 4 | 4 | 100% | 2 | 2 | 0 | 0% | 0 | 0 | Strong |
| Genus | Prevotella 2 | 1 | 1 | 100% | 1 | 0 | 0 | 0% | 0 | 0 | Weak |
| Genus | Prevotella 9 | 2 | 2 | 100% | 2 | 0 | 0 | 0% | 0 | 0 | Strong |
| Genus | Prevotellaceae NK3B31 group | 1 | 1 | 100% | 1 | 0 | 0 | 0% | 0 | 0 | Weak |
| Genus | Rhodoplanes | 1 | 1 | 100% | 1 | 0 | 0 | 0% | 0 | 0 | Weak |
| Genus | Romboutsia | 2 | 2 | 100% | 1 | 1 | 0 | 0% | 0 | 0 | Moderate |
| Genus | Roseburia | 4 | 4 | 83% | 1 | 3 | 0 | 0% | 0 | 0 | Moderate |
| Genus | Ruminiclostridium 5 | 2 | 0 | 0% | 0 | 0 | 2 | 100% | 1 | 1 | Moderate |
| Genus | Ruminococcaceae UCG 002 | 1 | 1 | 100% | 1 | 0 | 0 | 0% | 0 | 0 | Weak |
| Genus | Ruminococcaceae UCG-011 | 1 | 0 | 0% | 0 | 0 | 1 | 100% | 1 | 0 | Weak |
| Genus | Ruminococcaceae UCG-013 | 1 | 0 | 0% | 0 | 0 | 1 | 100% | 1 | 0 | Weak |
| Genus | Ruminococcaceae UCG-014 | 1 | 0 | 0% | 0 | 0 | 1 | 100% | 1 | 0 | Weak |
| Genus | Ruminococcus 2 | 1 | 0 | 0% | 0 | 0 | 1 | 100% | 1 | 0 | Weak |
| Genus | Ruminococcus torques group | 1 | 0 | 0% | 0 | 0 | 1 | 100% | 1 | 0 | Weak |
| Genus | Serratia | 1 | 1 | 100% | 1 | 0 | 0 | 0% | 0 | 0 | Weak |
| Genus | Shuttleworthia | 1 | 0 | 0% | 0 | 1 | 1 | 100% | 1 | 0 | Weak |
| Genus | Streptococcus | 4 | 0 | 0% | 0 | 0 | 4 | 100% | 2 | 2 | Strong |
| Genus | Synergistes | 2 | 2 | 100% | 2 | 0 | 0 | 0% | 0 | 0 | Strong |
| Genus | Thalassospira | 1 | 0 | 0% | 0 | 0 | 1 | 100% | 1 | 0 | Weak |
| Genus | Tyzzerella 3 | 2 | 2 | 100% | 1 | 1 | 0 | 0% | 0 | 0 | Moderate |
| Genus | Tyzzerella 4 | 1 | 0 | 0% | 0 | 0 | 1 | 100% | 1 | 0 | Weak |
| Genus | Veillonella | 1 | 0 | 0% | 0 | 0 | 1 | 100% | 1 | 0 | Weak |
| Species | Blautia obeum | 1 | 0 | 0% | 0 | 0 | 1 | 100% | 1 | 0 | Weak |
| Species | Clostridiales bacterium SIT11 | 1 | 0 | 0% | 0 | 0 | 1 | 100% | 1 | 0 | Weak |
| Species | Clostridium bolteae | 1 | 0 | 0% | 0 | 0 | 1 | 100% | 1 | 0 | Weak |
| Species | Clostridium citroniae | 1 | 0 | 0% | 0 | 0 | 1 | 100% | 1 | 0 | Weak |
| Species | Clostridium nexile | 1 | 1 | 100% | 1 | 0 | 0 | 0% | 0 | 0 | Weak |
| Species | Faecalibacterium prausnitzii | 3 | 3 | 100% | 1 | 2 | 0 | 0% | 0 | 0 | Moderate |
| Species | K. pneumoniae | 1 | 1 | 100% | 1 | 0 | 0 | 0% | 0 | 0 | Weak |
| Species | Lactobacillus plantarum | 1 | 1 | 100% | 1 | 0 | 0 | 0% | 0 | 0 | Weak |
| Species | Pseudomonas aeruginosa | 1 | 0 | 0% | 0 | 0 | 1 | 100% | 1 | 0 | Weak |
| Species | Roseburia intestinalis | 1 | 1 | 100% | 1 | 0 | 0 | 0% | 0 | 0 | Weak |
| Species | unclassified species (Escherichia) | 1 | 1 | 100% | 1 | 0 | 0 | 0% | 0 | 0 | Weak |
| Species | unclassified species (Parabacteroides) | 1 | 1 | 100% | 1 | 0 | 0 | 0% | 0 | 0 | Weak |
| OTU IDs | 194297 [Ruminococcaceae, Ruminococcus] | 1 | 1 | 100% | 1 | 0 | 0 | 0% | 0 | 0 | Weak |
| OTU IDs | 4483337 [Lachnospiraceae, Unclassified] | 1 | 1 | 100% | 1 | 0 | 0 | 0% | 0 | 0 | Weak |
| OTU IDs | Collapsed [Lachnospiraceae, Unclassified] | 1 | 1 | 100% | 1 | 0 | 0 | 0% | 0 | 0 | Weak |

*Level of evidence determined using the *system of evidence level* criteria.

^a^ Includes data from studies that investigated adults with CKD, IgAN, DN, ESKD, KT recipients and individuals who were receiving dialysis therapy (HD and PD).

**Table S2.** Dataset presenting the reported direction of microbial alteration in adults with kidney stones compared to controls.

| **Taxonomy** | **Microbiota** | **Total** | **Decreased findings** | | | | **Increased findings** | | | | **Level of evidence*** |
| --- | --- | --- | --- | --- | --- | --- | --- | --- | --- | --- | --- |
|  |  | Total no. studies reporting these taxa | No. Studies reporting decreased findings | Consistent percentage | Decreased findings (good quality) | Decreased findings (poor quality) | No. Studies reporting increased findings | Consistent percentage | Increased findings (good quality) | Increased findings (poor quality) | Rating |
| Phylum | Actinobacteria | 1 | 0 | 0% | 0 | 0 | 1 | 100% | 1 | 0 | Weak |
| Phylum | Actinobacteria | 1 | 0 | 0% | 0 | 0 | 1 | 100% | 1 | 0 | Weak |
| Phylum | Bacteroidetes | 2 | 2 | 100% | 1 | 1 | 0 | 0% | 0 | 0 | Moderate |
| Phylum | Fusobacteria | 1 | 0 | 0% | 0 | 0 | 1 | 100% | 1 | 0 | Weak |
| Class | Alphaproteobacteria | 2 | 0 | 0% | 0 | 0 | 2 | 100% | 2 | 0 | Strong |
| Class | Bacilli | 2 | 0 | 0% | 0 | 0 | 2 | 100% | 2 | 0 | Strong |
| Class | Bacteriodia | 1 | 1 | 100% | 1 | 0 | 0 | 0% | 0 | 0 | Weak |
| Class | Clostridia | 1 | 0 | 0% | 0 | 0 | 1 | 100% | 1 | 0 | Weak |
| Class | Coriobacteriia | 1 | 0 | 0% | 0 | 0 | 1 | 100% | 1 | 0 | Weak |
| Class | Epsilonproteobacteria | 1 | 0 | 0% | 0 | 0 | 1 | 100% | 1 | 0 | Weak |
| Class | Fusobacteriia | 1 | 0 | 0% | 0 | 0 | 1 | 100% | 1 | 0 | Weak |
| Class | Gammaproteobacteria | 1 | 0 | 0% | 0 | 0 | 1 | 100% | 1 | 0 | Weak |
| Class | Negativicutes | 1 | 0 | 0% | 0 | 0 | 1 | 100% | 1 | 0 | Weak |
| Order | Bacteroidales | 1 | 1 | 100% | 1 | 0 | 0 | 0% | 0 | 0 | Weak |
| Order | Bifidobacteriales | 1 | 0 | 0% | 0 | 0 | 1 | 100% | 1 | 0 | Weak |
| Order | Burkholderiales | 1 | 0 | 0% | 0 | 0 | 1 | 100% | 1 | 0 | Weak |
| Order | Caulobacteriales | 1 | 0 | 0% | 0 | 0 | 1 | 100% | 1 | 0 | Weak |
| Order | Clostridiales | 1 | 0 | 0% | 0 | 0 | 1 | 100% | 1 | 0 | Weak |
| Order | Coriobacteriales | 1 | 0 | 0% | 0 | 0 | 1 | 100% | 1 | 0 | Weak |
| Order | Erysipelotrichiales | 1 | 0 | 0% | 0 | 0 | 1 | 100% | 1 | 0 | Weak |
| Order | Fusobacteriales | 1 | 0 | 0% | 0 | 0 | 1 | 100% | 1 | 0 | Weak |
| Order | Lactobacillales | 1 | 0 | 0% | 0 | 0 | 1 | 100% | 1 | 0 | Weak |
| Order | Niesseriales | 1 | 0 | 0% | 0 | 0 | 1 | 100% | 1 | 0 | Weak |
| Order | Pasteurellales | 1 | 0 | 0% | 0 | 0 | 1 | 100% | 1 | 0 | Weak |
| Order | Selenomonadales | 1 | 0 | 0% | 0 | 0 | 1 | 100% | 1 | 0 | Weak |
| Family | Acidaminococcaceae | 1 | 0 | 0% | 0 | 0 | 1 | 100% | 1 | 0 | Weak |
| Family | Anaplasmataceae | 1 | 1 | 100% | 1 | 0 | 0 | 0% | 0 | 0 | Weak |
| Family | Bacteroidaceae | 1 | 1 | 100% | 1 | 0 | 0 | 0% | 0 | 0 | Weak |
| Family | Bacteroidales family S24-7 | 1 | 1 | 100% | 1 | 0 | 0 | 0% | 0 | 0 | Weak |
| Family | Bifidobacteriaceae | 1 | 0 | 0% | 0 | 0 | 1 | 100% | 1 | 0 | Weak |
| Family | Campylobacteraceae | 1 | 0 | 0% | 0 | 0 | 1 | 100% | 1 | 0 | Weak |
| Family | Caulobacteraceae | 1 | 0 | 0% | 0 | 0 | 1 | 100% | 1 | 0 | Weak |
| Family | Christensenellaceae | 1 | 1 | 100% | 1 | 0 | 0 | 0% | 0 | 0 | Weak |
| Family | Christensenellaceae R7 group | 1 | 1 | 100% | 1 | 0 | 0 | 0% | 0 | 0 | Weak |
| Family | Comamonadaceae | 1 | 0 | 0% | 0 | 0 | 1 | 100% | 1 | 0 | Weak |
| Family | Coriobacteriaceae | 1 | 0 | 0% | 0 | 0 | 1 | 100% | 1 | 0 | Weak |
| Family | Enterobacteriaceae | 2 | 0 | 0% | 0 | 0 | 2 | 100% | 1 | 1 | Moderate |
| Family | Enterococcaceae | 1 | 0 | 0% | 0 | 0 | 1 | 100% | 1 | 0 | Weak |
| Family | Fusobacteriaceae | 1 | 0 | 0% | 0 | 0 | 1 | 100% | 1 | 0 | Weak |
| Family | Lachnospiraceae | 1 | 0 | 0% | 0 | 0 | 1 | 100% | 1 | 0 | Weak |
| Family | Neisseriaceae | 1 | 0 | 0% | 0 | 0 | 1 | 100% | 1 | 0 | Weak |
| Family | Pasteurellaceae | 1 | 0 | 0% | 0 | 0 | 1 | 100% | 1 | 0 | Weak |
| Family | Rhodospirillaceae | 1 | 0 | 0% | 0 | 0 | 1 | 100% | 1 | 0 | Weak |
| Family | Rikenellaceae | 1 | 1 | 100% | 1 | 0 | 0 | 0% | 0 | 0 | Weak |
| Family | Streptococcaceae | 2 | 0 | 0% | 0 | 0 | 2 | 100% | 2 | 0 | Strong |
| Family | Veillonellaceae | 1 | 1 | 100% | 1 | 0 | 0 | 0% | 0 | 0 | Weak |
| Genus | Agromyces | 1 | 0 | 0% | 0 | 0 | 1 | 100% | 1 | 0 | Weak |
| Genus | Alistipes | 1 | 1 | 100% | 1 | 0 | 0 | 0% | 0 | 0 | Weak |
| Genus | Asteroleplasma | 1 | 1 | 100% | 1 | 0 | 0 | 0% | 0 | 0 | Weak |
| Genus | Asticcacaulis | 1 | 0 | 0% | 0 | 0 | 1 | 100% | 1 | 0 | Weak |
| Genus | Bifidobacterium | 1 | 0 | 0% | 0 | 0 | 1 | 100% | 1 | 0 | Weak |
| Genus | Blautia | 2 | 0 | 0% | 0 | 0 | 2 | 100% | 2 | 0 | Strong |
| Genus | Collinsella | 1 | 0 | 0% | 0 | 0 | 1 | 100% | 1 | 0 | Weak |
| Genus | Dialister | 1 | 0 | 0% | 0 | 0 | 1 | 100% | 1 | 0 | Weak |
| Genus | Dorea | 1 | 0 | 0% | 0 | 0 | 1 | 100% | 1 | 0 | Weak |
| Genus | Eisenbergiella | 1 | 0 | 0% | 0 | 0 | 1 | 100% | 1 | 0 | Weak |
| Genus | Enterococcus | 2 | 1 | 50% | 1 | 0 | 0 | 0% | 0 | 0 | Weak |
| Genus | Erysipelatoclostridium | 1 | 0 | 0% | 0 | 0 | 1 | 100% | 1 | 0 | Weak |
| Genus | Erysipelotrichaceae UCG 003 | 1 | 0 | 0% | 0 | 0 | 1 | 100% | 1 | 0 | Weak |
| Genus | Eubacterium ventriosum group | 1 | 1 | 100% | 1 | 0 | 0 | 0% | 0 | 0 | Weak |
| Genus | Faecalibacterium | 1 | 1 | 100% | 1 | 0 | 0 | 0% | 0 | 0 | Weak |
| Genus | Fusobacterium | 1 | 0 | 0% | 0 | 0 | 1 | 100% | 1 | 0 | Weak |
| Genus | Haemophilus | 1 | 0 | 0% | 0 | 0 | 1 | 100% | 1 | 0 | Weak |
| Genus | Herbaspirillum | 1 | 0 | 0% | 0 | 0 | 1 | 100% | 1 | 0 | Weak |
| Genus | Howardella | 1 | 0 | 0% | 0 | 0 | 1 | 100% | 1 | 0 | Weak |
| Genus | Lachnoclostridium | 1 | 0 | 0% | 0 | 0 | 1 | 100% | 1 | 0 | Weak |
| Genus | Lachnospiraceae UCG-004 | 1 | 1 | 100% | 1 | 0 | 0 | 0% | 0 | 0 | Weak |
| Genus | Lactobacillus | 1 | 0 | 0% | 0 | 0 | 1 | 100% | 1 | 0 | Weak |
| Genus | Megamonas | 1 | 1 | 100% | 1 | 0 | 0 | 0% | 0 | 0 | Weak |
| Genus | Neisseria | 1 | 0 | 0% | 0 | 0 | 1 | 100% | 1 | 0 | Weak |
| Genus | Niabella | 1 | 0 | 0% | 0 | 0 | 1 | 100% | 1 | 0 | Weak |
| Genus | Parabacteroides | 1 | 1 | 100% | 1 | 0 | 0 | 0% | 0 | 0 | Weak |
| Genus | Paraprevotella | 1 | 1 | 100% | 1 | 0 | 0 | 0% | 0 | 1 | Weak |
| Genus | Parasutterella | 1 | 0 | 0% | 0 | 0 | 1 | 100% | 1 | 0 | Weak |
| Genus | Pelomonas | 1 | 1 | 100% | 1 | 0 | 0 | 0% | 0 | 0 | Weak |
| Genus | Phascolarctobacterium | 1 | 0 | 0% | 0 | 0 | 1 | 100% | 1 | 0 | Weak |
| Genus | Prevotella | 1 | 1 | 100% | 1 | 0 | 0 | 0% | 0 | 0 | Weak |
| Genus | Prevotella 2 | 1 | 1 | 100% | 1 | 0 | 0 | 0% | 0 | 0 | Weak |
| Genus | Prevotella 9 | 1 | 1 | 100% | 1 | 0 | 0 | 0% | 0 | 0 | Weak |
| Genus | Romboutsia | 1 | 1 | 100% | 1 | 0 | 0 | 0% | 0 | 0 | Weak |
| Genus | Roseburia | 1 | 1 | 100% | 1 | 0 | 0 | 0% | 0 | 0 | Weak |
| Genus | Ruminococcaceae UCG 002 | 1 | 1 | 100% | 1 | 0 | 0 | 0% | 0 | 0 | Weak |
| Genus | Ruminococcus 2 | 1 | 0 | 0% | 0 | 0 | 1 | 100% | 1 | 0 | Weak |
| Genus | Ruminococcus torques group | 1 | 0 | 0% | 0 | 0 | 1 | 100% | 1 | 0 | Weak |
| Genus | Streptococcus | 2 | 0 | 0% | 0 | 0 | 2 | 100% | 2 | 0 | Strong |
| Genus | Subdoligranulum | 1 | 0 | 0% | 0 | 0 | 1 | 100% | 1 | 0 | Weak |
| Genus | Sutterella | 1 | 0 | 0% | 0 | 0 | 1 | 100% | 1 | 0 | Weak |
| Genus | Thalassospira | 1 | 0 | 0% | 0 | 0 | 1 | 100% | 1 | 0 | Weak |
| Genus | Tyzzerella 3 | 1 | 0 | 0% | 0 | 0 | 1 | 100% | 1 | 0 | Weak |
| Genus | Tyzzerella 3 | 1 | 1 | 100% | 1 | 0 | 0 | 0% | 0 | 0 | Weak |
| Genus | Veillonella | 1 | 0 | 0% | 0 | 0 | 1 | 100% | 1 | 0 | Weak |
| Species | Blautia obeum | 1 | 0 | 0% | 0 | 0 | 1 | 100% | 1 | 0 | Weak |
| Species | Clostridiales bacterium SIT11 | 1 | 0 | 0% | 0 | 0 | 1 | 100% | 1 | 0 | Weak |
| Species | Clostridium bolteae | 1 | 0 | 0% | 0 | 0 | 1 | 100% | 1 | 0 | Weak |
| Species | Clostridium citroniae | 1 | 0 | 0% | 0 | 0 | 1 | 100% | 1 | 0 | Weak |
| Species | Clostridium nexile | 1 | 1 | 100% | 1 | 0 | 0 | 0% | 0 | 0 | Weak |
| Species | Escherichia Shigella | 1 | 0 | 0% | 0 | 0 | 1 | 100% | 1 | 0 | Weak |
| Species | Faecalibacterium prausnitzii | 1 | 1 | 100% | 1 | 0 | 0 | 0% | 0 | 0 | Weak |
| Species | K. pneumoniae | 1 | 1 | 100% | 1 | 0 | 0 | 0% | 0 | 0 | Weak |
| Species | Lactobacillus plantarum | 1 | 1 | 100% | 1 | 0 | 0 | 0% | 0 | 0 | Weak |
| Species | Pseudomonas aeruginosa | 1 | 0 | 0% | 0 | 0 | 1 | 100% | 1 | 0 | Weak |
| Species | Roseburia intestinalis | 1 | 1 | 100% | 1 | 0 | 0 | 0% | 0 | 0 | Weak |
| Species | unclassified species (Parabacteroides) | 1 | 1 | 100% | 1 | 0 | 0 | 0% | 0 | 0 | Weak |

*Level of evidence determined using the *system of evidence level* criteria.

| **Table S3.** Presence and absence data of gene bearing species studied by the conserved genes such as 16S rRNA gene (archaea and eubacteria), 18S rRNA gene (microeukaryotes) and ITS region (fungi) sequencing summarised from Suryavanshi et al. 2018. | | | | | | | | |
| --- | --- | --- | --- | --- | --- | --- | --- | --- |
| **Microeukaryotes (18S rRNA gene sequencing)** | | | **Archaeal (16S rRNA gene sequencing)** | | | **Fungal (ITS region sequencing)** | | |
| **Absent^1^** | **Present^2^** | **Common^3^** | **Absent^1^** | **Present^2^** | **Common^3^** | **Absent^1^** | **Present^2^** | **Common^3^** |
| *Hanseniaspora guilliermondii* | *Arthrinium puccinioides* | *Blastocystis hominis* | *Halococcus dombrowskii* | *Methanosaeta concilii* | *Methanobrevibacter smithii* | *None reported* | *Botryosphaeria dothidea* | *Aspergillus niger* |
| *Foaina nana* | *Aspergillus penicillioides* | *Kazachstania viticola* | *Methanobacterium alcaliphilum* | *Methanoculleus palmolei* | *Methanobrevibacter gottschalkii* |  | *Lasiodiplodia theobromae* | *Aspergillus caesiellus* |
| *Syngamus trachea* | *Aureobasidium pullulans* |  | *Haloferax sulfurifontis* | *Methanofollis liminatans* | *Methanosphaera stadtmanae* |  | *theobromae* | *Macrophomina* |
| *Entamoeba dispar* | *Caenorhabditis plicata* |  |  | *Methanospirillum hungatei* | *Methanobrevibacter arboriphilus* |  | *Cladosporium cladosporioides* | *phaseolina* |
| *Boeremia exigua* | *Candida geochares* |  |  | *Methanoregula boonei* | *Methanobacterium bryantii* |  | *Phoma multirostrata* | *Coprinus comatus* |
|  | *Candida magnoliae* |  |  | *Methanobrevibacter millerae* | *Candidatus Nitrososphaera gargensis* |  | *Aspergillus candidus* | *Aspergillus sydowii* |
|  | *Candida orthopsilosis* |  |  | *Thermoplasma volcanium* | *Methanobrevibacter woesei* |  | *Aspergillus flavus* | *Saccharomyces cerevisiae* |
|  | *Candida piceae* |  |  | *Methanocorpusculum bavaricum* | *Methanobrevibacter ruminantium* |  | *Aspergillus fumigatus* |  |
|  | *Candida tropicalis* |  |  | *Methanobacterium sp. MB4* |  |  | *Aspergillus penicillioides* |  |
|  | *Davidiella tassiana* |  |  | *Haloarcula sinaiiensis* |  |  | *Emericella nidulans* |  |
|  | *Dioszegia crocea* |  |  | *Methanobacterium subterraneum* |  |  | *Eurotium amstelodami* |  |
|  | *Enteromonas hominis* |  |  | *Methanomethylovorans thermophila* |  |  | *Arthroderma benhamiae* |  |
|  | *Gibberella zeae* |  |  |  |  |  | *Kodamaea ohmeri* |  |
|  | *Kodamaea ohmeri* |  |  |  |  |  | *Pichia kudriavzevii* |  |
|  | *Lobophora variegata* |  |  |  |  |  | *Kluyveromyces marxianus* |  |
|  | *Metarhizium anisopliae* |  |  |  |  |  | *Candida inconspicua* |  |
|  | *Physalospora scirpi* |  |  |  |  |  |  |  |
|  | *Pichia kudriavzevii* |  |  |  |  |  |  |  |
|  | *Resinicium bicolor* |  |  |  |  |  |  |  |
|  | *Rhizopus stolonifer* |  |  |  |  |  |  |  |
|  | *Saccharomyces cerevisiae* |  |  |  |  |  |  |  |
|  | *Schistosoma mansoni* |  |  |  |  |  |  |  |
|  | *Sporisorium reilianum* |  |  |  |  |  |  |  |
|  | *Steinernema glaseri* |  |  |  |  |  |  |  |
|  | *Wickerhamiella domercqiae* |  |  |  |  |  |  |  |

^1^Absent in adults with kidney stones compared to controls; ^2^Present in adults with kidney stones compared to controls; ^3^Common to both adults with kidney stones and controls.
